# NLRX1 limits inflammatory neurodegeneration in the anterior visual pathway

**DOI:** 10.1186/s12974-025-03339-0

**Published:** 2025-01-28

**Authors:** Alexander J. Gill, Matthew D. Smith, Danny Galleguillos, Thomas Garton, Jackson W. Mace, Sachin P. Gadani, Swati Kumar, Aayush Pokharel, Krista Solem, Saahith Potluri, Omar Hussein, Giuliana Sardi Rogines, Arihant Singh, Annatje Clark, Peter A. Calabresi, Marjan Gharagozloo

**Affiliations:** 1 Department of Neurology, Division of Neuroimmunology, School of Medicine, Johns Hopkins University, Baltimore, MD 21287 USA; 2 Solomon H. Snyder Department of Neuroscience, Johns Hopkins University, Baltimore, MD 21205 USA

**Keywords:** MS, Innate immunity, NLRX1, Neurodegeneration, EAE, Retina, Optic nerve, Glia, Neuroinflammation

## Abstract

Chronic innate immune activation in the central nervous system (CNS) significantly contributes to neurodegeneration in progressive multiple sclerosis (MS). Using multiple experimental autoimmune encephalomyelitis (EAE) models, we discovered that NLRX1 protects neurons in the anterior visual pathway from inflammatory neurodegeneration. We quantified retinal ganglion cell (RGC) density and optic nerve axonal degeneration, gliosis, and T-cell infiltration in *Nlrx1*^*−/−*^ and wild-type (WT) EAE mice and found increased RGC loss and axonal injury in *Nlrx1*^*−/−*^ mice compared to WT mice in both active immunization EAE and spontaneous opticospinal encephalomyelitis (OSE) models. To minimize the effects of *Nlrx1*^*−/−*^ on peripheral lymphocyte priming during EAE, we performed adoptive transfer experiments, in which activated myelin-specific T cells were transferred into lymphocyte-deficient *Rag*^*−/−*^ or *Nlrx1*^*−/−*^*Rag*^*−/−*^ mice. In this model, we found more severe microgliosis and astrogliosis in the optic nerve of *Nlrx1*^*−/−*^*Rag*^*−/−*^ mice compared to *Rag*^*−/−*^ mice, suggesting a regulatory role of NLRX1 in innate immune cells. Transcriptome analysis in primary astrocytes activated with LPS and IFNγ demonstrated that NLRX1 suppresses NF-κB activation and regulates mitochondrial oxidative phosphorylation in inflammatory reactive astrocytes. The novel pharmacologic NLRX1 activators NX-13 and LABP-66 decreased LPS-mediated gene expression of inflammatory cytokines and chemokines in mixed glial cultures. Moreover, treating EAE mice with oral LABP-66, compared to vehicle, after the onset of paralysis resulted in less anterior visual pathway neurodegeneration. These data suggest that pharmacologic NLRX1 activators have the potential to limit inflammatory neurodegeneration. This study highlights that NLRX1 could serve as a promising target for neuroprotection in progressive MS and other neurodegenerative diseases. Further studies are needed to better understand the cell-specific mechanisms underlying the neuroprotective role of NLRX1 in response to inflammation in the CNS.

**Supplementary Information:**

The online version contains supplementary material available at 10.1186/s12974-025-03339-0.

## Introduction

Multiple sclerosis (MS) is an autoimmune disease of the central nervous system (CNS), characterized by chronic inflammation, gliosis, demyelination, and neurodegeneration [1]. The disease is commonly manifested by a relapsing-remitting (RR) phase at the early stage that frequently evolves into a progressive phase at the later stage. Current MS disease-modifying drugs mainly target peripheral immune cells that mediate RRMS and effectively control relapses. However, it remains a challenge to develop a treatment that limits the chronic inflammation in the CNS and prevents neurodegenerative processes that cause progressive MS (PMS) [2]. Understanding the mechanisms that drive PMS and identifying new therapeutic targets for PMS are a pressing unmet need.

Evidence suggests that a local breakdown in trophic support and anti-inflammatory cellular communication during the early stages of neurodegeneration plays a critical role in MS progression [3]. Disruptions in these essential interactions between neurons, glial cells, and immune cells can lead to an imbalance in homeostasis, triggering inflammatory cascades and loss of neuronal integrity. Inflammation-mediated neurodegeneration may result from direct effects on neurons or indirectly through glial-driven inflammation. Inflammatory responses in neurons can lead to intrinsic cell damage and death [4, 5], while activated glial cells, such as astrocytes and microglia, can release pro-inflammatory cytokines and neurotoxic factors that amplify neurodegeneration.

Recently, a neurodegenerative glial phenotype (‘microglia inflamed in MS’, MIMS; and ‘astrocytes inflamed in MS’, AIMS) was defined in chronically inflamed white matter lesions from postmortem MS brains [6], highlighting the significant contribution of inflammatory/neurotoxic glia in the progressive stages of MS. The nuclear factor kB (NF-κB) signaling pathway is an important proinflammatory signaling pathway and is highly activated in astrocytes and microglia during EAE and in MS active lesions [7–10]. These findings suggest that the treatment of progressive MS should be based on a combination of CNS anti-inflammatory and neuroprotective strategies.

Nucleotide binding and oligomeric domain-like receptor X1 (NLRX1) is an immunomodulatory protein of the NOD-like receptor (NLR) family that is located in mitochondria and widely expressed in all tissues [11, 12]. NLRX1 was initially identified in the setting of infection as a repressor of the pro-inflammatory type-I interferon [13–15] and canonical NF-κB [16, 17] immune pathways. Recent work has further identified a role for NLRX1 in modulating non-infectious inflammatory disease including limiting autoreactive T-cell responses [18], innate immune activation [19, 20], and oxidative stress-mediated tissue injury [21]. In autoimmune colitis models, *Nlrx1*^-/-^ mice exhibited more severe inflammatory disease with increased T-cell tissue infiltration, innate immune activation, and inflammatory cytokine release compared to wild-type (WT) mice [18]. Our group has previously shown that NLRX1 plays a key role in limiting tissue damage and controlling innate immune activation [22]. In the absence of NLRX1, these regulatory processes are unchecked, resulting in an increased incidence (~ 50%) of spontaneous EAE in 2D2 mice, which express transgenic T-cell receptors (TCRs) that recognize myelin-oligodendrocyte glycoprotein (MOG) [22]. Importantly, we discovered several gene variants within *NLRX1* that run in MS-affected families and are predicted to impair NLRX1 function [22]. However, the role of NLRX1 in molecular pathways related to glial inflammation and neuronal death in MS is poorly understood.

The purpose of this study was to investigate the role of NLRX1 in inflammatory neurodegeneration as occurs in progressive MS. We hypothesized that NLRX1 would be protective against inflammatory neurodegeneration. To test this, we used active, spontaneous, and adoptive transfer EAE models to define the impact of NLRX1 on inflammatory and neurodegenerative processes in the anterior visual pathway. The anterior visual pathway is an excellent location to more broadly model immune mediated neurodegeneration in MS as retinal pathology measured by optical coherence tomography (OCT) is associated with cortical and subcortical gray matter pathology in MS [23–26]. Additionally, the anatomy of the anterior visual pathway facilitates the assessment of reactive glia in the optic nerve, which are associated with axonal injury and subsequent retinal ganglion cell loss at the late stage of EAE [27, 28].

We further examined the potential therapeutic efficacy of activating NLRX1 using a small molecule NLRX1 agonist, LABP-66 (NX-64-3) [29], in limiting inflammation and neurodegeneration in the anterior visual pathway. In this work, we demonstrate that NLRX1 functions as a protective factor against RGC loss during active and spontaneous EAE and that LABP-66 can limit RGC neurodegeneration in active EAE. We found that NLRX1 has no effect on peripheral immune cell trafficking but does regulate the production of specific inflammatory mediators in the CNS including IL-1β and IL-6, which are elevated in the inflamed CNS. We further found that NLRX1 regulates the transcriptomic profile of astrocytes and that LABP-66 can limit inflammatory gene expression in reactive glia. Our results suggest that therapeutic targeting of NLRX1 using small molecule agonists limits inflammatory neurodegeneration and could be a potential therapeutic strategy for the compartmentalized CNS inflammation that occurs in progressive MS.

## Materials and methods

### Active EAE

C57BL/6J mice were purchased from The Jackson Laboratory. The animals were housed in the animal facility at the Johns Hopkins University School of Medicine 12 h/12 h light/dark cycles and fed with standard food and water. *Nlrx1*^*−/−*^ mice (C57BL/6J) were kindly provided by Dr. Denis Gris (Sherbrooke, QC, Canada) [20, 22]. We induced EAE in 9-week-old C57BL/6J WT and *Nlrx1*^*−/−*^ mice with myelin oligodendrocyte glycoprotein (MOG_35–55_) immunization as previously described [27]. Briefly, mice were immunized on day 0 subcutaneously at two sites over the lateral abdomen with an emulsion of MOG_35 − 55_ (200 µg, JHU Peptide Synthesis Core) in complete Freund’s adjuvant (CFA) containing 400 µg of *Mycobacterium tuberculosis* H37RA (Difco Laboratories). Pertussis toxin (250 ng; List Biological Labs, Campbell, CA, USA) was intraperitoneally injected on days 0 and 2. All immunized mice were weighed and scored daily using the established standard scoring scale from 1 to 5 as previously described [27]. Briefly, no disease signs = 0; loss of tail tonicity = 1; loss of tail tonicity and hindlimb paresis = 2; paralysis of hindlimbs = 3; hindlimb paralysis and forelimb paresis = 4; and complete paralysis or death = 5. Mice were sacrificed at post-immunization day (PID) 16 or PID 42, corresponding to the peak or late phase of EAE. All experimental protocols were performed in accordance with the National Institutes of Health guidelines for the use of experimental animals and were approved by the Johns Hopkins Institutional Animal Care and Use Committee.

### Opticospinal EAE (OSE)

2D2 TCR transgenic mice (TCR^MOG^) [30] were purchased from the Jackson Laboratory. Th mice (IgH^MOG^ or BCR^MOG^ mice) [31] were kindly provided by Dr. Scott Zamvil with permission from Dr. Hartmut Weckerle (Germany). These two transgenic mice lines were bred with *Nlrx1*^*−/−*^ mice and the triple transgenic mice (both male and female) were used for further experiments. Mice were monitored from weaning to 10-weeks of age. Body weight was measured and clinical EAE paralysis scores were recorded daily by a person blind to mouse genotype as described above [27]. Mice were sacrificed at 10 weeks old, and the tissues were collected for immunohistochemistry as described below.

### Adoptive transfer EAE

*Nlrx1*^*−/−*^ mice were crossed with *Rag2*^*−/−*^ C57BL/6J mice (Jackson Laboratory) to generate *Nlrx1*^*−/−*^*Rag*^*−/−*^ mice. T cells were isolated from TCR^MOG^ mice and polarized to Th17 in vitro using a previously published protocol [32]. Briefly, CD4^+^ T cells were isolated from the spleens and draining lymph nodes of TCR^MOG^ mice using the Mojosort CD4^+^ isolation kit (BioLegend) and co-cultured with irradiated WT splenocytes at a ratio of 1:5. Cells were cultured in the medium IMDM (Thermo 610 Fisher) supplemented with 10% fetal bovine serum (Gemini Bio-Products), 1% penicillin and streptomycin (Quality 611 Biologicals), 55µM 2-mercaptoethanol (Gibco), and 1% Glutamax (Thermo Fisher). We added the following reagents to the culture medium to polarize the T cells to Th17: anti-CD3 (2.5 µg/mL, BioLegend), anti-IL-4 (20 µg/mL, BioLegend), anti-IFN-γ (20 µg/mL, BioLegend), IL-6 (30 ng/mL, PeproTech), and TGFβ (3 ng/mL, Thermo Fisher). After 72 h incubation, cells were transferred to a new plate with fresh medium and 10 ng/mL IL-23 (R&D Systems). The cells were incubated in IL-23 for 48 h, then restimulated in the plate coated with anti-CD3 and anti-CD28 (2 µg/mL, BioLegend). After 48 h of restimulation, cells were collected, resuspended in PBS, and injected to *Rag2*^*−/−*^ or *Nlrx1*^*−/−*^*Rag*^*−/−*^ mice intraperitoneally (5 × 10^6^ Th17 cells, 200ul/mouse). The mice were monitored daily, and the weight and clinical paralysis scores were recorded. Fourteen days after adoptive transfer the mice were sacrificed, and tissues were collected for immunohistochemistry as described below. The details of cytokines and reagents used in the cell culture experiments are included in Suppl. Table 9.

### Flat mount retina and retinal ganglion cell counting

Mice were anesthetized with isoflurane gas and perfused transcardially with 50 ml PBS. The eyes were removed from the eye socket using curved forceps and immediately placed into 4% paraformaldehyde (PFA) for 4 h on the orbital shaker then transferred to 30% sucrose in PBS. Whole retinas were dissected from the eyes and retinal ganglion cells (RGCs) were stained as described previously [28]. Briefly, retinas were permeabilized with PBS containing 3% Triton X-100 and blocked with PBS containing 5% normal goat serum in PBS and 1% Triton X-100 for 1.5 h at room temperature with shaking. Sections were incubated with primary antibodies, anti-Brn3a antibody (Synaptic Systems #411 003, 1:1000) and pc-Jun (Cell Signaling Technology #3270, 1:1000), for 72 h at 4 °C with shaking. After washing with PBS, 3 times (for 1 h each), the sections were incubated with secondary antibody, Alexa Fluor 488 (Invitrogen #A11001, 1:1000) overnight at at 4 °C with shaking. After 3 washes with PBS, the sections were mounted using the Aqua-Poly/Mount reagent (Polysciences, Warrington, PA, USA). The whole-mount retinas were imaged using a Zeiss Axio Observer Z1 epifluorescence microscope. In each retina image, 12 regions (central, middle, and peripheral; 4 locations each) were selected and the number of RGCs was determined in the selected images in TIF format using a semi-automated MATLAB algorithm previously developed by our group [28]. The details of antibodies used for retina staining are included in Suppl. Table 10.

### Optic nerve and retina immunofluorescent staining

Mouse retina and optic nerves were collected as described previously [27]. After perfusion, the eyes were removed from the eye socket using curved forceps and immediately placed into 4% PFA for 4 h and then transferred to 30% sucrose in PBS until the tissue sank.

For vertical sectioned retinas, the eyes were placed into TissueTek mold filled with Optimal Cutting Temperature (O.C.T.) compound (VWR, Radnor, PA, USA), snap frozen on dry ice and then stored at − 80 °C until cryosectioned. The eyes were sectioned at 16 μm vertically and then mounted onto slides (ThermoScientific, Rockford, IL, USA) followed by staining with antibodies. Optic nerves were dissected out and evenly divided into three parts and then vertically placed into TissueTek mold filled with O.C.T. and snap frozen on dry ice. Frozen samples were stored at − 80 °C until cryosectioning. Optic nerves were sectioned at 16 μm thickness and then mounted on slides for further immunofluorescence staining.

For immunostaining, we washed the sections with PBS with 0.4% of Triton X-100 (3 times, 5 min each) then blocked with blocking buffer (PBS with 5% normal goat or donkey serum and 0.4% Triton X-100) for one hour at room temperature. Primary antibodies were incubated with optimized concentrations overnight at 4 °C. The details of antibodies used for staining optic nerve are included in Suppl. Table 10. The next day, after 3 washes with PBS with 0.1% of Triton X-100, species-specific secondary antibodies directly conjugated to Alexa fluorophores were incubated with sections for 1 h at room temperature. After this incubation, sections were washed, and nuclear staining was performed with Hoechst. Sections were mounted with a coverslip using ProLong™ Glass Antifade Mountant (Invitrogen) and imaged using using a Zeiss Axio Observer Z_1_ epifluorescence microscope with the appropriate excitation and emission filters. Three sections were stained and analyzed from the anterior, middle, and posterior regions of the optic nerve per animal. Fiji-ImageJ (National Institutes of Health) were used for the quantification of the markers.

### Western blot

Mice were perfused with cold PBS and spinal cords were dissected immediately and flash-frozen in liquid N2. For immunoblotting, samples were homogenized in ice-cold RIPA buffer (50mM Tris-HCl pH 8, 150mM NaCl, 1% IGEPAL CA-630, 0.5% sodium deoxycholate, 0.1% SDS, 1X cOmplete protease inhibitor (Roche 04693116001), 1X Halt Phosphatase Inhibitor (Thermo Scientific 1862495), using a Dounce homogenizer. Samples were centrifuged at 21,000 g for 15 min at 4 °C. Protein concentration in the supernatants was measured using the bicinchoninic acid (BCA) assay (Thermo). Thirty micrograms of proteins were separated by electrophoresis on a 4–20% SDS polyacrylamide gel and transferred onto Immobilon-FL polyvinylidene fluoride (PVDF) membrane (Millipore). All membranes were blocked with Intercept Blocking Buffer and incubated overnight at 4 °C with primary antibodies, followed by the appropriate IRDye secondary antibody (1:20,000, LI-COR Biotechnology) for 45 min at RT. Infrared signal was acquired and quantified using the Odyssey Imaging System. The details of primary antibodies used for Western blot are shown in the Suppl. Table 10.

### Flow cytometric analysis of CNS-infiltrating mononuclear cells

Mice were euthanized with isoflurane and then perfused transcardially with PBS without cations. The spinal cords were extracted by flushing them from the vertebral column using hydrostatic pressure. The tissue was mechanically dissociated and enzymatically digested using collagenase IV (0.5 mg/ml, Worthington) and DNAse I (100 U/ml, Worthington) and then passed it through a 100-µm nylon sieve. Subsequently, cells were separated from myelin debris using debris removal solution (Miltenyi Biotec) and the cellular pellet was collected. The cells were stained with viability dye and antibodies against surface markers and run on a Cytek Aurora cytometer. The data were analyzed with the FlowJo software. The details of antibodies and reagents used for flow cytometry staining are shown in the Suppl. Table 11.

### Primary astrocyte culture

Astrocytes were isolated from brains of P6 mouse pups (C57/BL6J WT and *Nlrx1*^−/−^). After dissecting off the meninges, whole brains underwent mechanical trituration and enzymatic dissociation (Papain 20U/ml; DNAse 100 U/mL) over 30 min at 37^o^C, followed by equal volume addition of 1% BSA in HBSS (+/+), centrifugation (5 min, 200 g), resupension in 0.02% BSA HBSS (+/+), and filtration through 100 μm strainer. Cells were Fc blocked and positively selected using the mouse anti-ACSA-2 MicroBead Kit (Miltenyi Biotec #130-097-678) and LS Columns per manufacturer’s instruction and then seeded onto poly-D-lysine-coated 6-well plates at a density of 5.0 × 10^5^ cells/well. Astrocytes were cultured in serum free media containing 50% DMEM, 50% neurobasal, 1% penicillin/streptomycin, N-acetyl cysteine (5 µg/mL), and 1× SATO, and supplemented with heparin-binding EGF (HB-EGF) (5 ng/mL, PeproTech) as previously described [33]. Astrocytes were grown for approximately 10 days until cultures reached a desired density, with media changes every 2 days. Cells were then treated with vehicle (H_2_O) or 500 ng/ml lipopolysaccharide (LPS; Millipore Sigma) and 10 ng/ml IFNγ (Peprotech). After 24 h cell lysates were collected in RLT buffer followed by RNA extraction using the RNeasy Plus Mini Kit (Qiagen). The purity of primary astrocyte cultures was confirmed by immunofluorescence staining of cells with GFAP and Sox9 as astrocyte markers and Iba1 as a microglia marker. Briefly, cells were seeded in a 24-wells plate with 1 × 10 mm coverslip. After washing with ice-cold PBS, cells were fixed with 4% PFA at 4 °C for 20 min on ice. Cells were washed twice with ice-cold PBS and the incubated with 500 µL of 0.15% Triton X100 on ice for 15 min. After washing the coverslips with ice-cold PBS and blocking with 10% normal goat serum at 4 °C for 20 min, primary antibodies were added and incubated overnight at 4 °C. Coverslips were then washed with PBS and incubated with secondary antibodies in blocking buffer for 1 h at room temperature. Next, the coverslips were washed with PBS and incubated with Hoeschst 33,342 solution (Invitrogen, H3570). Coverslips were washed and mounted onto glass slides using Aqua poly/mount (Polysciences, 18606-2). The details of antibodies used to confirm astroglia purity are shown in Suppl. Table 10.

### Primary mouse mixed glial culture and drug treatment

Brains of P1-P2 mouse pups (C57/BL6J WT and *Nlrx1*^*−/−*^*)* were dissected and mechanically and enzymatically dissociated as above. Equal volume of mixed-glia media (DMEM/F12, 10% FBS, 1mM sodium-pyruvate, 1% penicillin/streptomycin, and 50 μm beta-mercaptoethanol) was added to single cell suspensions followed by centrifugation (5 min, 200 g) and resuspension of cell-pellet in mixed-glia media. Cells were plated in poly-L-lysine coated T75 flasks (1 brain/flask) and cultured with 50% media changes every 3 days. After 2 weeks in culture, cells were transferred using trypsin-digestion to poly-L-lysine coated 12-well plates (1 flask/plate) and mixed-glia media. After 1 day, media was fully exchanged with serum-free DMEM/F12 containing either vehicle (0.01% DMSO), 1µM NX-13, or 200nM LABP-66 (Landos Biopharma). Twenty-four hours later mixed-glia cultures were stimulated with either vehicle (PBS) or 250 ng/ml LPS (Cell Signaling). Twenty-four hours after stimulation cell lysates were collected in RTL buffer followed by RNA extraction using the RNeasy Plus Mini Kit (Qiagen) and cDNA generation using the iScript cDNA Synthesis Kit (Bio-Rad).

### Bulk RNA-seq

Isolated RNA from astrocyte cultures was submitted to NovoGene who prepared poly-A capture RNA-seq libraries in non-strand specific fashion followed by sequencing of each library (~ 30 M paired end 150 bp reads per library). Transcripts were quantified with Salmon [34] (v1.7.0) using selective alignment with a decoy-aware index generated from the GRCm39 transcriptome using the full genome as a decoy [35]. Percent alignments had an average of 88.12% with a minimum of 84.8%. The transcript-level quantifications were then imported into R (v4.3.2) with tximport [36] (v1.30.0) and condensed to the gene level. Libraries were then analyzed with two separate Bioconductor pipelines - DESeq2 [37] (v1.42.1) and Limma-Voom [38] (v3.58.1) following package authors recommendations [36]. The design formula for the differential expression testing was: *~0 + genotype + genotype: litter + genotype: condition* which allowed us to test for an interaction of genotype and condition (Vehicle vs. LPS + IFNγ) while blocking for biological replicates (litter) within each genotype. For DESeq2, transcript quantifications were converted into DESeqDataSet object with function *DESeqDataSetFromTximport()* then we ran the *DESeq()* function with default settings. Results were extracted for the interaction of genotype and condition as well as comparing just *Nlrx1*^*−/−*^ vs. WT conditions with the *results()* function. For the purposes of hypergeometric testing, genes were considered to be differentially expressed if their adjusted p.value was less than 0.05. All lists of differentially expressed genes were generated with DESeq2 (Suppl. Tables 1, 2, 3). For limma-voom, length scaled counts generated by *tximport* were converted into a DGEList object. Low expressed genes were filtered out using *filterByExpr()* with a group size of 4. Scaling factors were calculated using the Trimmed Mean of M-values (TMM) method with the *calcNormFactors()* function. Data was then transformed with *voom()*, a linear model for each gene fitted with *lmFit()*, coefficients and standard errors computed with *contrasts.fit()*, and finally moderated t-statistics and a moderated F-statistic were generated with *eBayes()*, all using default options. Competitive gene set testing comparing how *Nlrx1*^*−/−*^ astrocytes responded to LPS + IFNγ stimulation differently than WT ([*Nlrx1*^*−/−*^ Stimulated – *Nlrx1*^*−/−*^ Unstim] compared to [WT Stimulated – WT Unstim]) was performed with the CAMERA algorithm [39], a competitive gene set test, on the EList object created by *voom()* as described above with default settings. The Mouse Ortholog Hallmark Gene Sets (from the Molecular Signatures Database (MSigDb) [40, 41] were used as the gene sets to be tested. The full CAMERA results are in Suppl. Table 2. The barcode plots in Fig. 5 and Suppl. Fig. 7 were generated by the limma function *barcodeplot() * and are arranged by the moderated t statistic generated by *eBayes()* in the limma-voom pipeline in increasing order. Genes on the left most portion of the x-axis, which have more negative moderated t statistics, are more likely to be differentially expressed in the negative direction (i.e. downregulated) while those on the right of the x-axis have larger moderated t statistics and are more likely to be differentially expressed in the positive direction (i.e. upregulated). The enrichment worm in the barcode plots is generated by the *tricubeMovingAverage()* function from the limma package to show local enrichment in each area of the x-axis. The heatmaps in Fig. 5 and Suppl. Fig. 7 are normalized log 2-fold change for each biological replicate (stimulated vs. unstimulated) as calculated by limma-voom then scaled by gene (row). The hierarchical clustering dendrograms in the heatmaps were calculated using the Manhattan distances and average method in R using the base stats package. The heatmaps were generated with pheatmap (v1.0.12). Hypergeometric testing for gene ontology (GO) terms [42, 43] (Suppl. Fig. 8, Suppl. Tables 4–8) was performed with clusterProfiler [44] (v4.10.1) using differentially expressed genes from the DESeq2 pipeline analysis with the universe defined as all genes in the GRCm39 transcriptome reference that were matched to the GO database (*n* = 22,345). Only the Biological Processes ontology was tested. The GO terms were sourced from the org.Mm.eg.db package [45] (v3.18.0) with a GO source date of 2023-07-27.

### Quantification of gene expression by qPCR

Cells were collected directly in RLT buffer followed by RNA extraction using the RNeasy Plus Mini Kit (Qiagen). For spinal cord tissue, mice were perfused with cold PBS, the spinal cords were dissected and immediately flash-frozen in liquid N2. RNA was extracted using RNeasy Plus Micro Kit (Qiagen). In both cases, cDNA was generated using the iScript cDNA Synthesis Kit (Bio-Rad). Levels of target gene expression in cDNA samples were determined using iQ SYBR Green Supermix (Bio-Rad) and gene specific primers (Integrated DNA Technologies), as detailed in Suppl. Table 12, on the CFX384 Real-Time System (Bio-Rad). For primary cultures, the Expression levels of the target genes were normalized to β-actin gene (*Actb*) and HPRT gene expression for primary cultures and spinal cord tissue, respectively. Fold changes over control conditions were calculated using the 2^−ΔΔCt^ method.

### LABP-66 treatment of EAE mice

Active EAE was induced in C57BL/6J female mice, which were then randomized to receive daily gavage of LABP-66 20 mg/kg (Landos Biopharma) [29] or vehicle (0.5% methylcellulose) on the day they developed a clinical paralysis score of 1.0 or greater. Mice were clinically scored daily by a blinded rater. Mice were sacrificed and the retinas and optic nerves were collected at PID 42.

### Data collection and statistical analysis

Blinding and randomization were performed for each study presented. EAE scoring, RGC quantification, and image analysis were performed in a blinded fashion. For experiments with treatment paradigms, namely the LABP-66 experiments, mice were matched based on initial EAE score on day of treatment start and then randomized. Statistical analyses were completed using GraphPad Prism 10.1.2. Statistical comparisons of continuous numerical variables were presented as mean ± standard error of the mean (SEM) and were analyzed by unpaired or paired Student’s T-test for two groups and by one-way ANOVA with appropriate post-hoc testing when comparing more than two groups. Data was assessed for normalcy before being assessed with Student t-tests. Statistical comparisons of discrete non-linear numerical variables, such as EAE scores, were presented as median ± interquartile range (IQR) and were analyzed by non-parametric Mann–Whitney *U* test. Statistical comparisons of proportions were analyzed by Fisher’s exact test. Correlations between continuous numerical variables were analyzed by Pearson correlation. *P*-values < 0.05 were considered statistically significant.

## Results

### NLRX1 deficiency is associated with enhanced neuroinflammation at the peak EAE

**Fig. 1:**
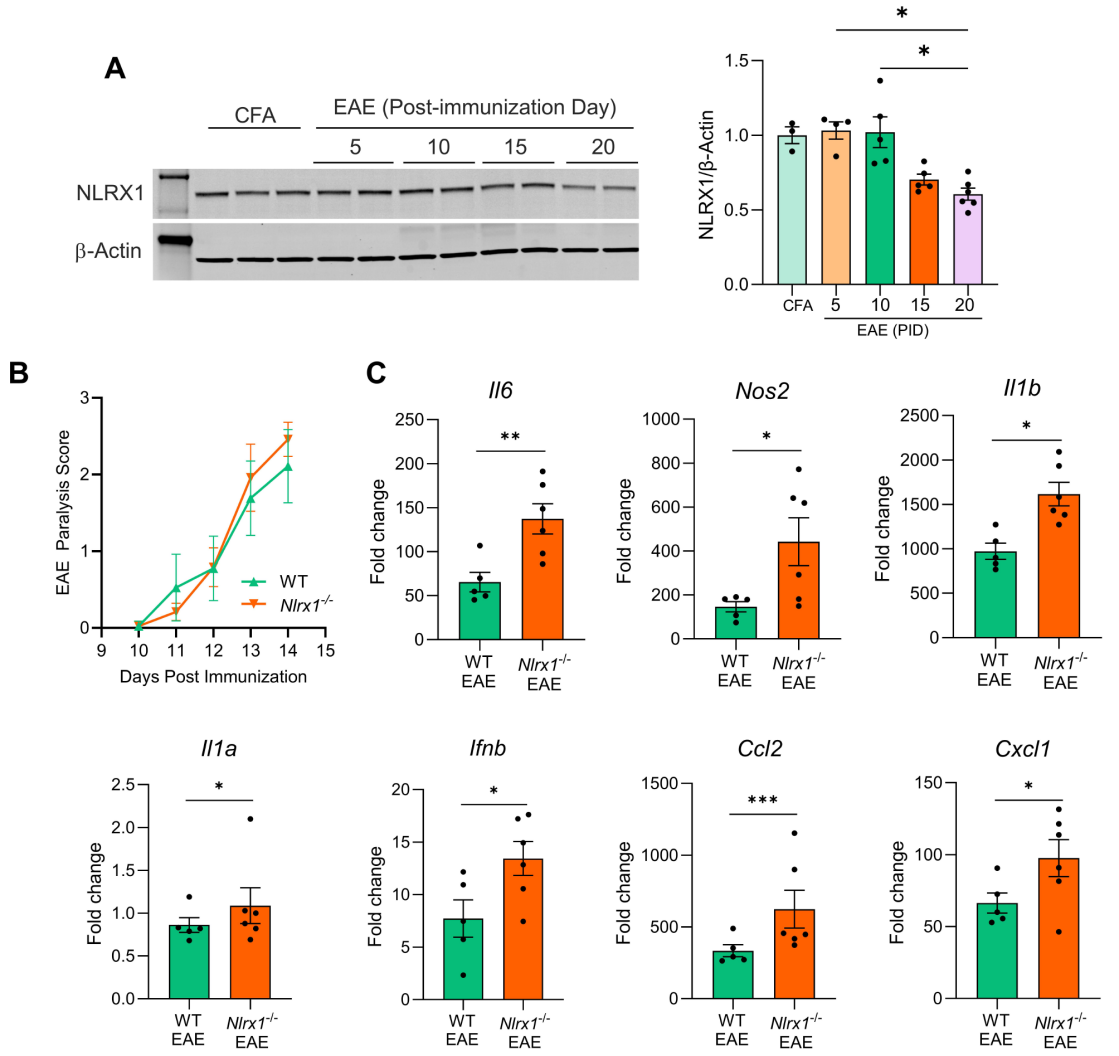
NLRX1 expression and inflammatory mediators during acute EAE. **(A)** Representative Western blot and densitometry analysis of NLRX1 protein expression in the spinal cord of CFA-treated (*n* = 3) and EAE mice (PID 5, 10, 15 and 20; *n* = 4–6). **(B)** WT and *Nlrx1*^-/-^ mice were immunized with MOG_35 − 55_ and their paralysis score was evaluated every day until tissue collection for RNA extraction. **(C)** Gene expression analysis of proinflammatory mediators in the spinal cord of WT (*n* = 5) and *Nlrx1*^-/-^  (*n* = 6) mice at PID14. Gene expression was normalized to CFA treated mice (data not shown). Data presented as mean at ± SEM. Western blot data was compared using one-way ANOVA with Holm-Sidak’s multiple comparison test. Gene expression data was analyzed using Fisher’s LSD test. *p < 0.05, ***p < 0.001, ****p < 0.0001

Investigating NLRX1 in the EAE model is crucial for understanding its role in neurodegeneration, particularly within the context of MS, where inflammation and neurodegeneration are intricately linked. MS is characterized by chronic inflammation, demyelination, and progressive neuronal damage, and the EAE model replicates many of these pathological features, especially in the spinal cord and optic nerve. Previous studies have demonstrated that NLRX1 plays a key role in regulating inflammation in various preclinical models. We first sought to evaluate whether NLRX1 expression might be influenced by inflammatory conditions in the CNS. To test this, we immunized WT mice with the (MOG)_35–55_ peptide to induce active EAE and quantified NLRX1 protein levels in the spinal cord by Western blot (Fig. 1A). We found that NLRX1 remains unchanged during the preclinical stage on post-immunization days (PID) 5 and PID 10. However, NLRX1 protein levels begin to decline by PID15 and drop to its lowest point at PID 20, during the peak of EAE. For this analysis, we utilized an NLRX1-specific antibody, whose specificity was validated using spinal cord samples from *Nlrx1*^*−/−*^ naïve and EAE mice as controls (Suppl. Fig. 1A). To further investigate the impact of NLRX1 deficiency on inflammation, we induced EAE in WT and *Nlrx1*^*−/−*^ mice and recorded daily clinical score until day 14, when the mice were sacrificed and the lumbar spinal cord was collected to assess the expression of inflammatory mediators at the peak of EAE (PID14). Although there was no significant difference in clinical scores between WT and *Nlrx1*^*−/−*^ mice (Fig. 1B), our qPCR analysis revealed a significant increase in a specific subset of inflammatory genes, including *Il6*,* Nos2*,* Il1a*,* Il1b*,* Ifnb*,* Ccl2*,* and Cxcl1* in *Nlrx1*^*−/−*^ EAE mice compared to WT EAE mice (Fig. 1C). However, the expression *of Tnf*,* Ifnb*, and *Ccl20* was similar between *Nlrx1*^*−/−*^ and WT EAE mice at this timepoint (Suppl. Fig. 1B). Additionally, when we measured the expression of *Iba1*,* Gfap*, and several inflammatory markers associated with reactive astrocytes, such as *Lcn2*,* Ligp1*,* C3*,* and Psmb8*, we observed no significant differences (Suppl. Fig. 1B, C). To evaluate whether the trafficking of immune cells into the spinal cord differed between the groups, we performed flow cytometry and found no significant differences in immune cell infiltration (T cells and myeloid cells) to the spinal cord of *Nlrx1*^*−/−*\n^ and WT EAE mice at peak EAE (Suppl. Fig. 1D, E). These findings suggest that, despite the differences in a specific subset of inflammatory mediators, the overall immune cell recruitment and markers of glial activation were comparable between *Nlrx1*^*−/−*^ and WT EAE mice at the peak of inflammation.

### NLRX1 protects RGCs against inflammatory neurodegeneration in active EAE

**Fig. 2:**
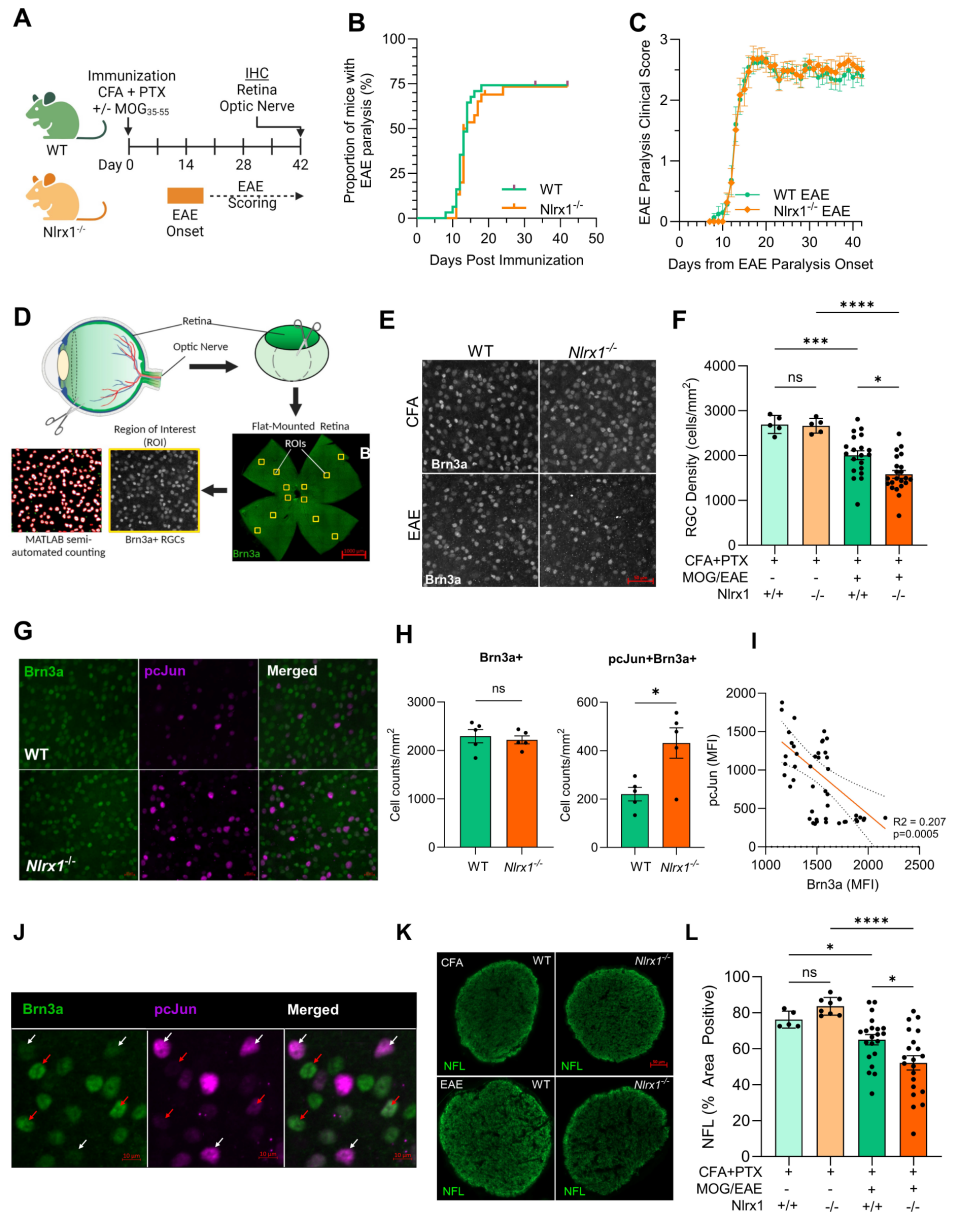
Clinical disease and anterior visual pathway degeneration in WT and *Nlrx1*^*−/−*^ active EAE mice. (**A**) WT and *Nlrx1*^*−/−*^ mice were immunized with subcutaneous injections of MOG_35 − 55_ in complete Freund’s adjuvant (CFA) and intraperitoneal injection of pertussis toxin (PTX). Control WT and *Nlrx1*^*−/−*^ mice received CFA and PTX without MOG_35 − 55_ (CFA-only controls). Mice were clinically monitored for (**B**) EAE onset and (**C**) EAE paralysis severity overtime (*n* = 22 WT, *n* = 23 *Nlrx1*^*−/−*^). EAE paralysis score data represents mean ± standard error of the mean (SEM). On day 42 post-immunization (PID 42) mice were euthanized, and eyes and optic nerves were collected for immunofluorescence staining (IF). **(D)** Retinas were dissected and flat-mounted for IHC and Brn3a + retinal ganglion cells (RGCs) were quantified in 12 regions of interests (ROIs) using a MATLAB semi-automated counting algorithm. Each RGC density/count data point corresponds to an individual mouse, showing the averaged counts from 12 regions in the retina (4 central, 4 middle, and 4 peripheral). (**E**) Representative IF images of Brn3a^+^ RGCs in flat-mounted retinas from CFA-only and EAE WT and *Nlrx1*^*−/−*^ mice. (**F**) Average RGC density for EAE (*n* = 20 WT, *n* = 22 *Nlrx1*^*−/−*^) and control mice, (CFA-controls = 10, 5 WT and 5 *Nlrx1*^*−/−*^). **(G)** Representative IF images of Brn3a + RGCs and phosphorylated (Ser73) c-Jun (pc-Jun) expression in flat-mounted retinas from WT and *Nlrx1*^−/−^ EAE mice. **(H)** Density quantification of total Brn3a + RGCs and pc-Jun + Brna3 + RGCs in WT and *Nlrx1*^−/−^ EAE mice. **(I)** Correlation between pc-Jun and Brna3 intensity in RGCs from WT and *Nlrx1*^−/−^ EAE mice. The mean intensity of each marker in a single RGC was measured in retina using Zeiss ZEN software. **(J)** Representative IF staining of wholemount retina from an *Nlrx1*^*−/−*^ EAE mouse, demonstrating pc-Jun^low^Brn3a^high^ RGCs (red arrows) and pc-Jun^high^Brn3a^low^ RGCs (white arrows). **(K)** Representative IF images of neurofilament light chain (NFL) and **(L)** average NFL percent area positive in the optic nerve of EAE and control mice. RGC density and NFL percent area positive data are represented as mean ± SEM with statistical analysis by Student’s t-test or one-way ANOVA with Dunnett’s T3 multiple comparison test. Linear associations were determined by Pearson correlation analysis. *p < 0.05, ***p < 0.001, ****p < 0.0001

Given that we observed elevated expression of potentially harmful inflammatory mediators, including IL-6, inducible nitric oxide synthase (iNOS), and IL-1β in *Nlrx1*^*−/−*^ EAE mice at the peak of inflammation, we hypothesized that the absence of NLRX1 would result in exacerbated tissue injury and worsened neurodegeneration during the chronic stage of EAE. To test this hypothesis, we focused on the anterior visual pathway, where optic nerve inflammation and RGC loss during EAE can be clearly and reliably quantified. Our approach has been validated for reproducibility, and we have published findings showing that inflammatory/reactive astrocytes mediate RGC loss in the retina at the chronic stage of EAE [27, 28, 46]. We immunized WT and *Nlrx1*^*−/−*^ mice against MOG_35 − 55_ peptide to induce active EAE and recorded the daily behavioral scores and the weight of mice for 42 days (Fig. 2A). Consistent with our previous finding (Fig. 1B), there was no difference in the proportion of mice that developed paralysis, the timing of paralysis onset post immunization, or the severity of tail and hind-limb paralysis between WT and *Nlrx1*^*−/−*^ mice (Fig. 2B,C), all of which are secondary to acute peripheral inflammatory infiltrate into the spinal cord [47].

Forty-two days post immunization, a chronic EAE timepoint, mice were euthanized and retinas and optic nerves were isolated to assess neurodegeneration in the anterior visual pathway. Retinas were dissected and flat-mounted, and Brn3a-positive RGCs were identified by immunofluorescence staining (IF) and counted semi-automatedly using a MATLAB algorithm across 4 representative regions of central, middle, and peripheral retina (12 regions total) (Fig. 2D). As we previously reported [27, 28], we observed a significant reduction in RGC density in WT EAE mice compared to control non-EAE WT mice that received only adjuvants without the MOG_35 − 55_ peptide (Fig. 2E,F). The RGC loss was significantly worsened in *Nlrx1*^*−/−*^ EAE mice compared to WT EAE mice (59.5% greater reduction). Importantly, we observed no significant difference in RGC density between WT and *Nlrx1*^*−/−*^ adjuvant-only control mice (Fig. 2F), demonstrating that loss of NLRX1 does not alter RGC density independently of the inflammation associated with EAE induction. The RGC loss was only significant at the late stage of EAE, no difference in RGC density was observed between naïve mice or EAE mice at the peak stage of EAE (Suppl. Fig. 2A). We assessed potential sex differences in RGC loss and observed that *Nlrx1*^−/−^ female mice exhibited significantly more severe RGC loss compared to males, relative to CFA controls (Suppl. Fig. 2B). In males, RGC loss was also present in *Nlrx1*^*−/−*^ mice but was less pronounced and not statistically significant compared to WT males. Clinical EAE scores were analyzed separately for males and females, and no differences were found between the sexes (Suppl. Fig. 2C).

Given the increased loss of RGCs in *Nlrx1*^−/−^ mice in late-stage EAE, we wanted to assess the cellular stress status of RGCs at an acute timepoint, before Brn3a+ RGC loss is observed (Fig. 2G,H). To accomplish this, we quantified Brn3a and phosphorylated (Ser73) c-Jun (pc-Jun) at the peak of EAE (PID16). The expression of pc-Jun is implicated in axonal injury-induced RGC death and is considered as an indicator of neuronal stress [48–50]. At this timepoint, we found twice (203%) as many pc-Jun + RGCs in *Nlrx1*^−/−^ EAE mice compared to WT mice (Fig. 2G,H), suggesting a higher percentage of RGCs (19.5% vs. 9.6%, respectively) that are at risk for future neurodegeneration. The expression level of Brn3a has been shown to decrease in stressed, damaged, and neurodegenerating RGCs [51]. Consistent with this, we observed a significant negative association between pc-Jun and Brn3a expression intensity within individual RGCs (Fig. 2I). These findings highlight that despite no observed difference in peripheral immune infiltration and activation, RGCs have increased cellular stress in *Nlrx1*^−/−^ EAE mice compare to WT EAE mice at the peak of EAE.

To assess the axonal integrity of RGCs, we quantified the expression of neurofilament light chain (NFL) in the optic nerve of the EAE mice using IF) [52]. We observed a decreased expression of NFL in the optic nerve of *Nlrx1*^*−/−*^ EAE compared to WT EAE group (Fig. 2K,L), which is consistent with the changes of RGC density in these mice (Fig. 2F). As expected, RGC density in the retina and NFL staining in the optic nerve were significantly positively correlated (Suppl. Fig. 2D). In addition, we observed a greater reduction in synapse integrity in the retinal inner-plexiform layer in *Nlrx1*^*−/−*^ EAE mice compared to WT EAE mice, consistent with their heightened neuronal injury in the anterior visual pathway (Suppl. Fig. 2E, F).

To investigate whether immune cell infiltration contributed to these changes, we performed immunofluorescence staining for T cells (CD3 + cells) and glial markers (Iba1 for microglia/myeloid cells and GFAP for astrocytes) in the optic nerve. We found no significant differences in immune cells or gliosis between WT and *Nlrx1*^*−/−*^ EAE mice (Suppl. Fig. 2G, 2H). To investigate whether the exacerbated RGC loss observed in *Nlrx1*^*−/−*^ EAE mice was due to increased infiltration of peripheral immune cells into the optic nerve at the earlier stage of the disease, we performed immunofluorescence staining on optic nerve sections from WT and *Nlrx1*^*−/−*^ mice at peak disease (PID16). We examined T cells (CD3+ cells) to evaluate lymphocytic infiltration, Iba1 expression to assess the presence and activation of myeloid cells and microglia, GFAP to quantify astrocyte activation, and myelin basic protein (MBP) to evaluate myelin integrity. Our analysis revealed no significant differences in the density of CD3+ T cells, Iba1 + myeloid cells/microglia, or GFAP + astrocytes between WT and *Nlrx1*^*−/−*^ EAE mice in the optic nerve. Additionally, MBP staining showed no detectable differences in myelin integrity between the two groups (Suppl. Fig. 3A–D). We also assessed GFAP and Iba1 expression in the retina of EAE mice at the peak stage of the disease and found no difference between WT and *Nlrx1*^−/−^ mice (Supp. Fig. 3E, F).

### NLRX1 protects RGCs against inflammatory neurodegeneration in OSE mice

**Fig. 3:**
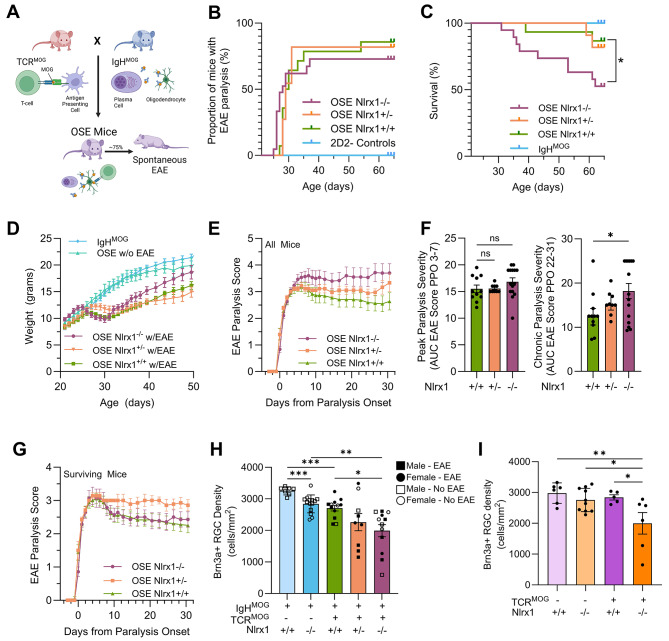
EAE clinical severity and anterior pathway neurodegeneration in *Nlrx1*^−/−^ opticospinal encephalomyelitis (OSE) mice. **(A)** OSE mice have T-cells that express a transgenic T-cell receptor (TCR^MOG^) and B-cells that express transgenic antibodies (IgH^MOG^), both of which recognize MOG_35 − 55_ peptide. **(B)** Paralysis time of onset, incidence, and **(C)** survival, **(D)** weight, and **(E)** EAE paralysis score of all WT (*n* = 14), *Nlrx1*^*+/−*^ (*n* = 11), and *Nlrx1*^*−/−*^ (*n* = 21) OSE mice and IgH^MOG^-only mice (*n* = 26). **(F)** Area under the curve of EAE paralysis score at peri-peak (days 3–7 post-paralysis onset/PPO) and chronic time points (22–31 PPO). **(G)** EAE paralysis scores over time and **(H)** Brn3^+^ RGC density of surviving WT, *Nlrx1*^*+/−*^, and *Nlrx1*^*−/−*^ OSE mice (excludes scores of 5.0). Square and circle data points identify male and female mice, respectively. Filled in and open data points identify mice that developed clinical EAE and those that did not, respectively. (**I**) Brn3a^+^ RGC density in mice with WT TCR (*n* = 16) and TCR^MOG^ mice with either *Nlrx1*^*+/+*^ (*n* = 5) or *Nlrx1*^*−/−*^ (*n* = 6). Averages are presented as mean ± SEM with statistical analysis performed by one-way ANOVA with post hoc Dunnett’s multiple comparison test. *p < 0.05, **p < 0.01, ***p < 0.001

Since NLRX1 acts as a regulatory molecule, and the regulatory effects of NLRs can vary based on the type and magnitude of immunological challenge [53], we wanted to examine the neuroprotective role of NLRX1 in a spontaneous EAE model, in which the mice develop EAE without any experimental intervention. We used the opticospinal encephalomyelitis (OSE) model, a spontaneous genetic EAE model [54], in which B-cells express a transgenic immunoglobulin that recognizes MOG (IgH^MOG^), and T-cells express a transgenic T-cell receptor that recognize MOG (TCR^MOG^) (Fig. 3A). We have previously shown that in our vivarium TCR^MOG^ X IgH^MOG^ or OSE mice develop spontaneous ascending paralysis or classical EAE approximately 75% of the time [46], while IgH^MOG^ mice do not develop paralysis, and TCR^MOG^ mice rarely develop classical EAE (0–4%, depending on the lab) [30, 46]. To assess the role of NLRX1 in this spontaneous EAE model, we generated *Nlrx1*^*−/−*^, *Nlrx1*^*+/−*^, and *Nlrx1*^*+/+*^ OSE littermates along with IgH^MOG^ littermate controls. There was no significant difference in age of EAE onset, proportion of mice that developed EAE, or changes in weight between these three OSE lines (Fig. 3B,D). However, there was a significant decrease in survival in *Nlrx1*^*−/−*^ OSE mice compared to *Nlrx1*^*+/+*^ OSE mice (Fig. 3C; Suppl. Fig. 4A), suggesting loss of NLRX1 enhanced disease pathology in this model. When including all OSE mice that developed EAE, there were significantly higher EAE paralysis scores at chronic, but not acute or peak, time points as measured by area under the curve (Fig. 3E,F). However, this analysis includes mice that died or needed to be euthanized, which equates to a EAE paralysis score of 5. When only mice that survived until the end of the experiment were analyzed, there was no significant difference in paralysis scores between *Nlrx1*^*−/−*^ and *Nlrx1*^*+/+*^ OSE mice (Fig. 3G).

Despite this disproportional loss of the clinically sickest *Nlrx1*^*−/−*^ OSE mice, the surviving *Nlrx1*^*−/−*^ OSE mice still showed a greater decrease in RGC density compared to *Nlrx1*^*+/+*^ OSE mice (Fig. 3H). In contrast to MOG_35 − 55_ immunization EAE, we observed that OSE mice, even those that did not develop clinically apparent paralysis, had significant decreases in RGC density, suggesting the presence of subclinical spontaneous optic neuropathy in these mice. Additionally, we observed an unexpected significant decrease in RGC density in IgH^MOG^ *Nlrx1*^*−/−*^ control mice compared to IgH^MOG^ *Nlrx1*^*+/+*^ control mice (Fig. 3H), suggesting that in the setting of NLRX1 loss and associated immune dysregulation, anti-MOG immunoglobulins can lead to some degree of optic neuropathy. We evaluated potential sex differences in survival and RGC loss in OSE mice and found no significant sex effect. Both male and female *Nlrx1*^*−/−*^ mice showed reduced survival and greater RGC loss compared to controls (Suppl. Fig. 4B, C).

We observed a similar phenomenon in TCR^MOG^ mice that did not develop clinically apparent EAE symptoms. TCR^MOG^ *Nlrx1*^*−/−*^ mice showed a significant loss of RGCs compared to TCR^MOG^ *Nlrx1*^*+/+*^ mice (Fig. 3I), suggesting that in the absence of NLRX1, MOG-specific T-cells can also lead to optic neuropathy independent from spinal cord pathology.

### NLRX1 regulates glial activation in the optic nerve during adoptive transfer EAE

**Fig. 4:**
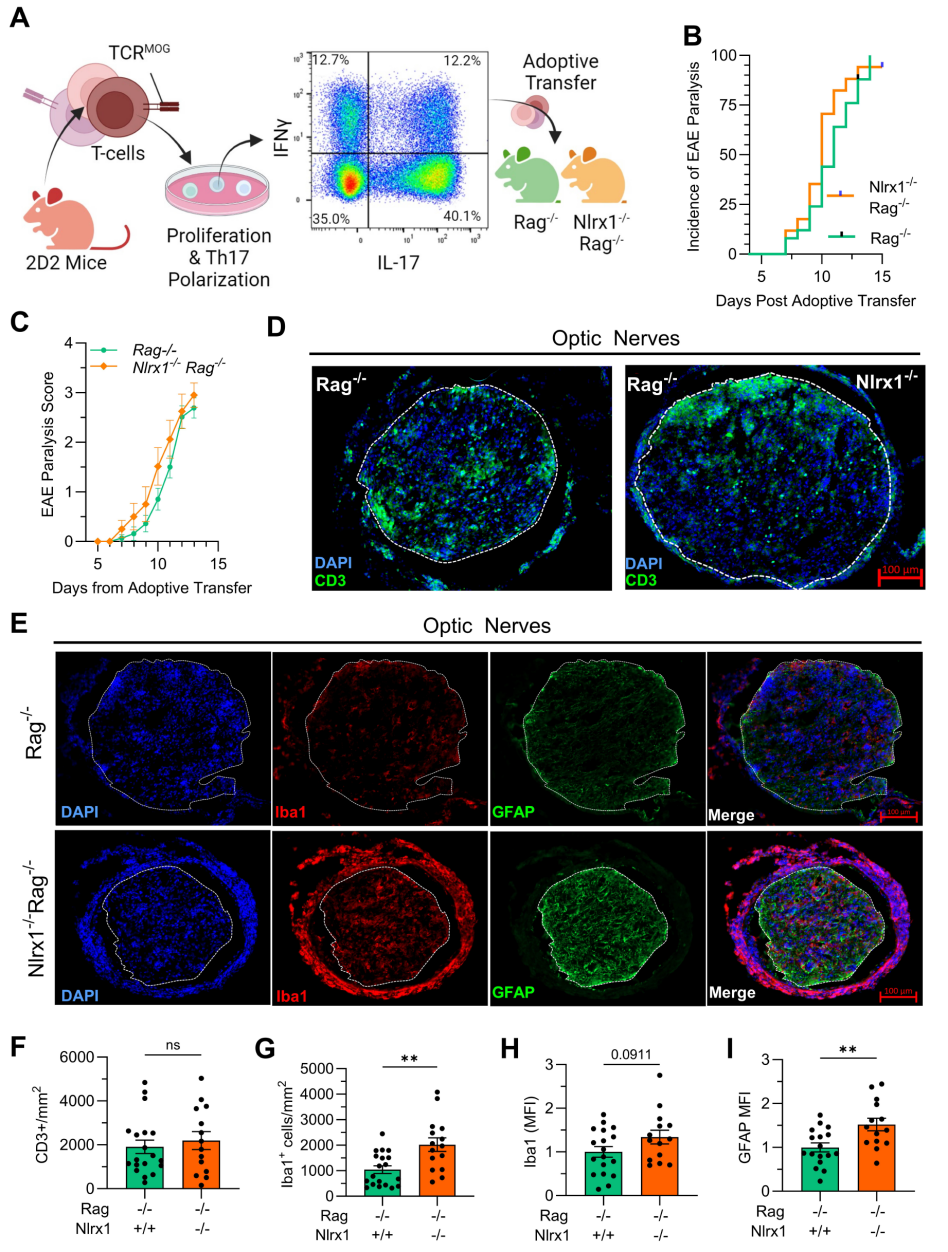
T-cell infiltration and glial activation in the optic nerve of *Rag*^*−/−*^ and *Nlrx1*^*−/−*^ *Rag*^*−/−*^ mice after adoptive transfer of activated anti-MOG CD4 + T-cells. (A) CD4 + T-cells are isolated from TCR^MOG^ mice and are proliferated, stimulated, and Th17 polarized in vitro. Subsequently these activated anti-MOG T-cells are injected intraperitoneally into Rag^−/−^ and *Nlrx1*^*−/−*^;*Rag*^*−/−*^ recipient mice. (B) Paralysis time of onset and incidence; and (C) paralysis severity by EAE clinical score in *Rag*\n^−/−^ (*n* = 25) and *Nlrx1*^*−/−*^ *Rag*^*−/−*^ (*n* = 17) recipient mice. Optic nerves were isolated from adoptively transferred mice two weeks after injection of anti-MOG T-cells and proximal, medial, and distal segments were analyzed by IHC for CD3, IBA1, and GFAP. Representative immunofluorescence (IF) images of cross-sectional optic nerves for DAPI and (D) CD3 and (E) IBA1 and GFAP in *Rag*^*−/−*^ (*n* = 17–19) and *Nlrx1*^*−/−*^ *Rag*^*−/−*^ (*n* = 14) recipient mice that developed EAE. Optic nerve and region of interest for quantification outlined with a dotted white line. Quantification of (F) CD3^+^ T-cell infiltration, (G) Iba1^+^ cell density, (H) Iba1 mean fluorescent intensity (MFI), and (I) GFAP MFI in *Rag*^*−/−*^ and *Nlrx1*^*−/−*^ *Rag*^*−/−*^ recipient mice. Data are presented as mean ± SEM with statistical analysis performed by Student’s t-test. **p < 0.01

NLRX1 has been shown to regulate T cell responses by inhibiting T cell proliferation and differentiation into Th1 and Th17 subsets [18, 22]. To isolate the specific role of NLRX1 in innate immunity, independent of its effects on T cell responses, we performed an adoptive transfer experiment using lymphocyte-deficient *Nlrx1*^*−/−*^*Rag*^*−/−*^ mice [22]. We isolated T-cells from TCR^MOG^ mice and after polarizing to Th17 cells in vitro, we transferred them intraperitoneally into *Rag*^*−/−*^ or *Nlrx1*^*−/−*^*Rag*^*−/−*^ mice (Fig. 4A). Comparing *Rag*^*−/−*^ or *Nlrx1*^*−/−*^*Rag*^*−/−*^ recipient mice after adoptive transfer, we observed no difference in the time of EAE onset, incidence, or severity (Fig. 4B, C; Suppl. Fig. 5A). Mice were euthanized two weeks after adoptive transfer at peak of EAE clinical onset to evaluate neurodegeneration and immune cell infiltration. Consistent with active EAE at the peak stage, we quantified RGCs and found no differences at this acute inflammatory timepoint between *Rag*^*−/−*^ and *Nlrx1*^*−/−*^*Rag*^*−/−*^ EAE mice (Suppl. Fig. 5B).

To assess immune infiltration and glial activation, we focused on parenchymal expression of markers including CD3+, Iba1+, and GFAP, as these activated cells are in direct contact with the RGC axons that run through the optic nerve. At this acute time point, there was no significant difference in T-cell infiltration in the optic nerve between *Rag*^*−/−*^ or *Nlrx1*^*−/−*^*Rag*^*−/−*^ recipient mice (Fig. 4D). However, we observed a significant increase in the density of Iba1+ cells and a trend towards an increase in Iba1 mean fluorescence intensity (MFI), indicative of activated microglia/macrophages. We also found a higher MFI for GFAP, a marker of astrocyte reactivity, in the optic nerve of *Nlrx1*^*−/−*^*Rag*^*−/−*^ mice compared to *Rag*^*−/−*^ mice (Fig. 4E-I). To validate these findings, we assessed immune cell infiltration into the brain of adoptive transfer EAE mice using flow cytometry and observed no significant differences in the inflammatory infiltrate within the brain at the peak EAE (PID14) between *Rag*^*−/−*^ or *Nlrx1*^*−/−*^*Rag*^*−/−*^ recipient mice (Suppl. Fig. 5 C, D). The observed increase in the density and activation of microglia (Iba1+ cells) and reactive astrocytes (GFAP expression) in the adoptive transfer EAE model highlights a potential role for NLRX1 as a regulator of inflammatory responses in glial cells. To further explore this hypothesis, we next investigated how NLRX1 in astrocytes affects their transcriptomic profile under inflammatory conditions in vitro.

### NLRX1 regulates the transcriptome of reactive astrocytes

**Fig. 5:**
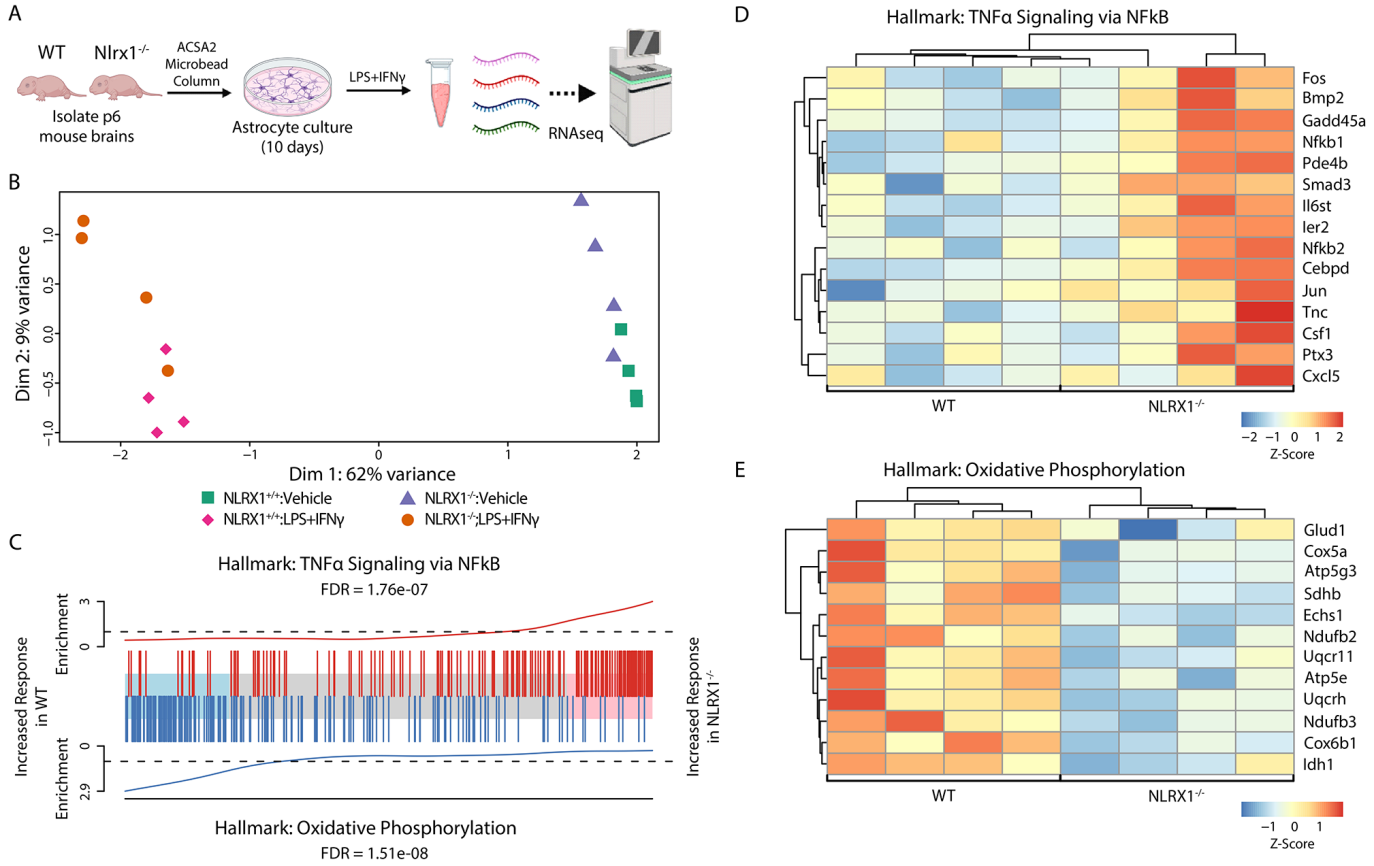
Transcriptome in LPS- and IFNγ-stimulated WT and *Nlrx1*^−/−^ primary astrocytes. **(A)** Schematic of the experimental design. Primary astrocyte cultures were derived from WT and *Nlrx1*^*−/−*^ mice and were then subsequently stimulated with IFNγ and lipopolysaccharide (LPS) or vehicle (phosphate buffered saline) for 24 h at which point RNA was isolated and analyzed by bulk RNA sequencing. **(B)** Multidimensional scaling (MDS) plot of the top 1000 highly variable genes from normalized RNA-seq libraries from *Nlrx1*^+/+^ and *Nlrx1*^−/−^ astrocyte cultures treated with or without LPS + IFNγ **(C)** Barcode plot showing genes associated with TNFα Signaling Via NF-κB (top, red) or genes associated with Oxidative Phosphorylation (bottom. blue). Genes associated with the pathway in question are represented by solid vertical line and are ranked from left to right using the moderated t statistic based on the difference in how *Nlrx1*^*−/−*^ cells responded to LPS + IFNγ vs. *Nlrx1*^*+/+*^. Genes toward the left had a higher induction in the WT than in *Nlrx1*^−/−^ while genes on the right had a higher induction in the *Nlrx1*^−/−^ mice. Dotted lines represent neutral enrichment, and solid lines represent local enrichment in that area of the ranked list generated by a weighted tricube average in a sliding window, so that a value of 1 represents enrichment if distribution of genes were uniform, and a value of 1.5 would mean a 50% increased enrichment within the window. FDR are false discovery rate values calculated by the competitive gene set test CAMERA. **(D and E)** Heatmap showing scaled log 2-fold change values (LPS + IFNγ  stimulated vs. unstimulated) where row represents a gene, and column represents an independent biological replicate for a selection of genes associated with **(D)** TNFα Signaling via NF-κB and **(E)** Oxidative Phosphorylation. Each column’s genotype is indicated below the heatmap. for each gene and each biological replicate

To further investigate whether NLRX1 directly regulates astrocyte inflammatory activation, we prepared primary astrocyte cultures from WT and *Nlrx1*^*-/-*^ pups (P0-P1) born on the same day to ensure comparable conditions. We performed immunofluorescent staining using DAPI as a nuclear marker, GFAP and Sox9 as astrocyte-specific markers, and Iba1 to assess the purity of isolated astrocytes. Our results showed that 92.5% and 96.3% of Dapi + cells were positive for GRAP and Sox9 respectively, with no identifiable Iba1 + positive cells (Suppl. Fig. 6). We subsequently stimulated these cultures with LPS and IFNγ or left them unstimulated for 24 h and then performed bulk RNAseq (Fig. 5A). When comparing stimulated WT and *Nlrx1*^*-/-*^ astrocytes, the difference between samples was primarily driven by the effect of LPS + IFNγ inflammatory signals. However, we also saw consistent differences between the normal and *Nlrx1*^*-/-*^ astrocytes in both treated and untreated conditions (Fig. 5B). Next, we looked specifically at how *Nlrx1*^*-/-*^ astrocytes responded differently to the LPS + IFNγ treatment compared to WT astrocytes ([stimulated *Nlrx1*^*-/-*^ – unstimulated *Nlrx1*^*-/-*^] compared to [stimulated WT – unstimulated WT]). Using the threshold of adjusted p.value < 0.05 to define differentially expressed genes, we identified that 125 genes were more upregulated, and 235 genes were downregulated in the *Nlrx1*^*-/-*^ astrocytes compared to the WT cells when treated with LPS + IFNγ (Suppl. Table 1).

When we tested different gene sets, we found that certain pathways, like TNFα signaling via NF-κB (Fig. 5C,D) and the Unfolded Protein Response, were more strongly activated in *Nlrx1*^*−/−*^ astrocytes (Suppl. Table 2). In contrast, pathways such as Oxidative Phosphorylation (Fig. 5C,E) and E2F Targets were less active in these *Nlrx1*^*−/−*^ astrocytes compared to WT astrocytes (Suppl. Fig. 7). Genes included in the Hallmark TNFα signaling via NF-κB (Fig. 5D) include NF-κB-pathway transcription factors (e, g. *NF-κB1*,* NF-κB2*) and several genes previously implicated in regulating reactive astrocyte phenotypes, including *Smad3*, *Bmp2*, *Cepbd*, and *Tnc* [55–60]. The oxidative phosphorylation pathway (Fig. 5E) included multiple subunits of each major mitochondrial respiratory chain complex (e.g. Ndufb* genes, Complex I/NADH Dehydrogenase; Sdh* genes, Complex II; Uqcr* genes, Complex III; Cox* genes, Complex IV; Atp5* genes, Complex IV/Mitochondrial ATP Synthase) and other enzymatic genes such as *Idh1* (isocitrate dehydrogenase) and *Glud1* (glutamate dehydrogenase).

When comparing gene expression of *Nlrx1*^*−/−*^ vs. WT astrocytes in either vehicle (*Nlrx1*^*−/−*^ Unstim – WT Unstim) or LPS + IFNγ conditions (*Nlrx1*^*−/−*^ LPS + IFNγ – WT LPS + IFNγ), we found enrichment of similar gene ontology terms associated with ribonucleoprotein complex biogenesis, cytoplasmic translation, RNA splicing, and nuclear cytoplasmic transport in the genes upregulated in *Nlrx1*^*−/−*^ vs. WT, while gene ontology terms associated with regulation of neurogenesis, gliogenesis, and autophagy were enriched in the downregulated genes (Suppl. Fig. 8; Suppl. Tables 1–8). The similarity of the pathways enriched in both the unstimulated and LPS + IFNγ conditions suggest these are the effects caused by *Nlrx1* depletion independent of inflammatory stimulation.

### NLRX1 agonists reduce inflammatory gene expression in reactive glial cells

**Fig. 6:**
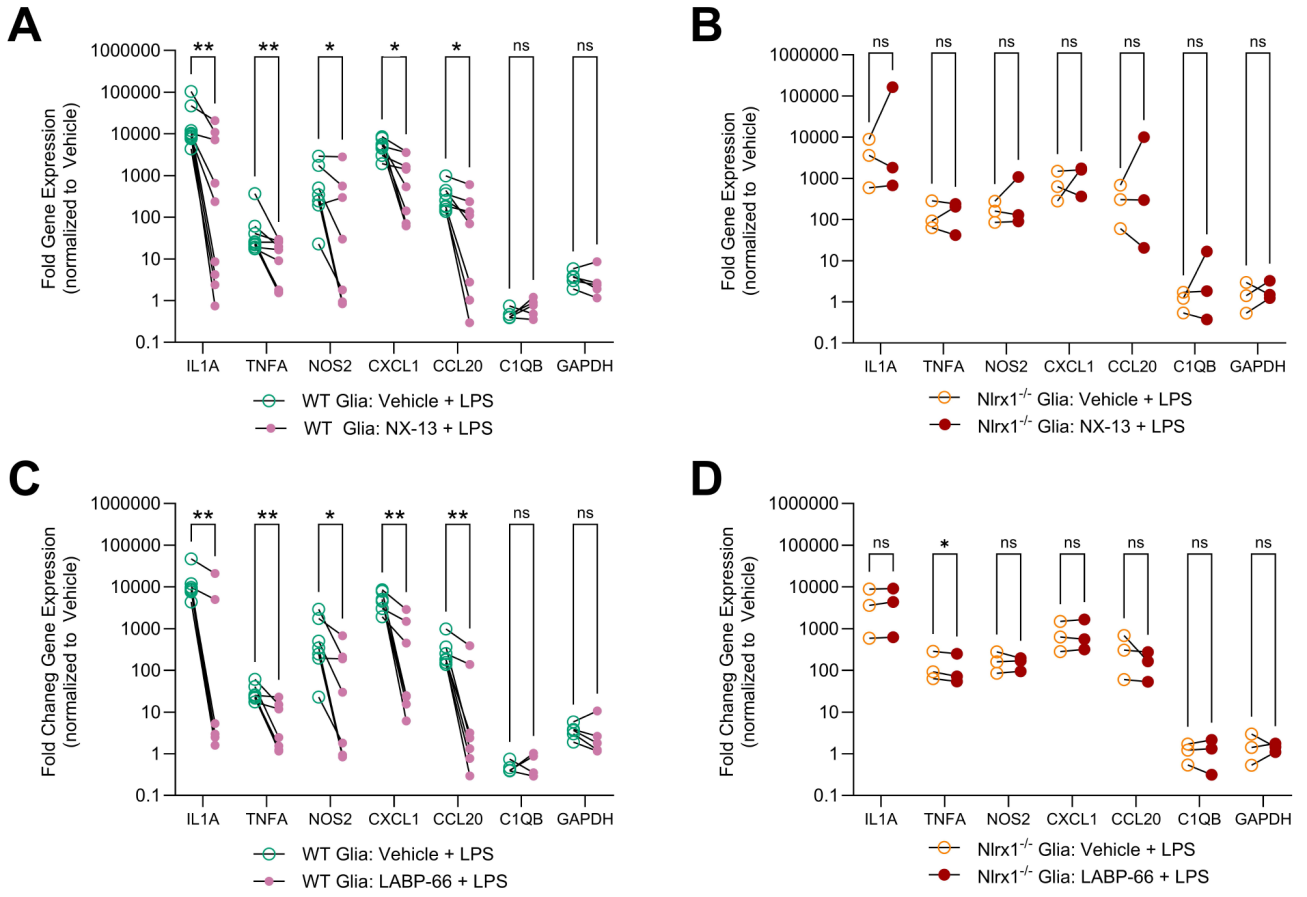
Inflammatory gene expression in LPS-stimulated primary mixed glial cultures. Primary mixed glial cultures were generated from WT and *Nlrx1*^−/−^ pups and cultured in vitro. Mixed glia was pre-treated with either 1µM of NX-13, 200 nm of LABP-66, or vehicle (0.01% DMSO) and were subsequently stimulated with 250ng/ml of LPS or vehicle (PBS). After 24 h of stimulation, mixed glial cultures RNA was isolated for subsequent gene expression analysis by quantitative PCR. Gene expression of select NF-κB-regulated and control genes in NX-13 pre-treated **(A)** WT (*n* = 5–7) and **(B) ***Nlrx1*^*−/−*^ (*n* = 3) glia and LABP-66 pre-treated **(C)** WT (*n* = 5–7) and **(D) ***Nlrx1*^*−/−*^ (*n* = 3) glia. Gene expression in glia pre-treated with vehicle is represented by open circles while gene expression in glia pre-treated with an NLRX1 activator is represented by closed circles. Gene expression is presented as fold expression normalized to vehicle treated, unstimulated mixed glia. Solid lines connect paired samples from the same biological replicate. Statistical analysis was performed by paired Student’s t-test. *p < 0.05, **p < 0.01, ***p < 0.001

Given the observed association between the loss of NLRX1 expression and increased glial activation, we hypothesized that enhancing NLRX1 activity in glial cells could attenuate their inflammatory response. To test this hypothesis, we used two recently identified NLRX1 activators, NX-13 and LABP-66. These compounds were developed through medicinal chemistry-based optimization of novel scaffolds designed to target the previously characterized binding pocket of NLRX1, thereby stabilizing NLRX1 [61–63], with LABP-66 specifically designed to be brain-penetrant [64]. We pretreated WT or *Nlrx1*^*−/−*^ primary mouse mixed glial cells with either NX-13 or LABP-66 before stimulating them with LPS to induce TLR4-dependent NF-κB activation. Twenty-four hours post-LPS stimulation, pretreatment with NX-13 and LABP-66 resulted in significantly lower expression of several NF-κB regulated genes, including *Il1a*, *Tnf*, *Nos2*, *Cxcl1*, and *Ccl20* in WT glia (Fig. 6A,C). In contrast, in *Nlrx1*^*−/−*^ glia we did not observe a significant reduction in these inflammatory genes with pre-treatment with NX-13 or LABP-66 (Fig. 6B, D). These findings suggest that the NLRX1 activators NX-13 and LABP-66 effectively inhibit inflammatory glial cells in an NLRX1-dependent manner. Next, we explored whether pharmacological activation of NLRX1 in vivo could protect RGCs from inflammatory degeneration associated with chronic EAE.

### LABP-66 ameliorates paralysis and RGC neurodegeneration in active EAE

**Fig. 7:**
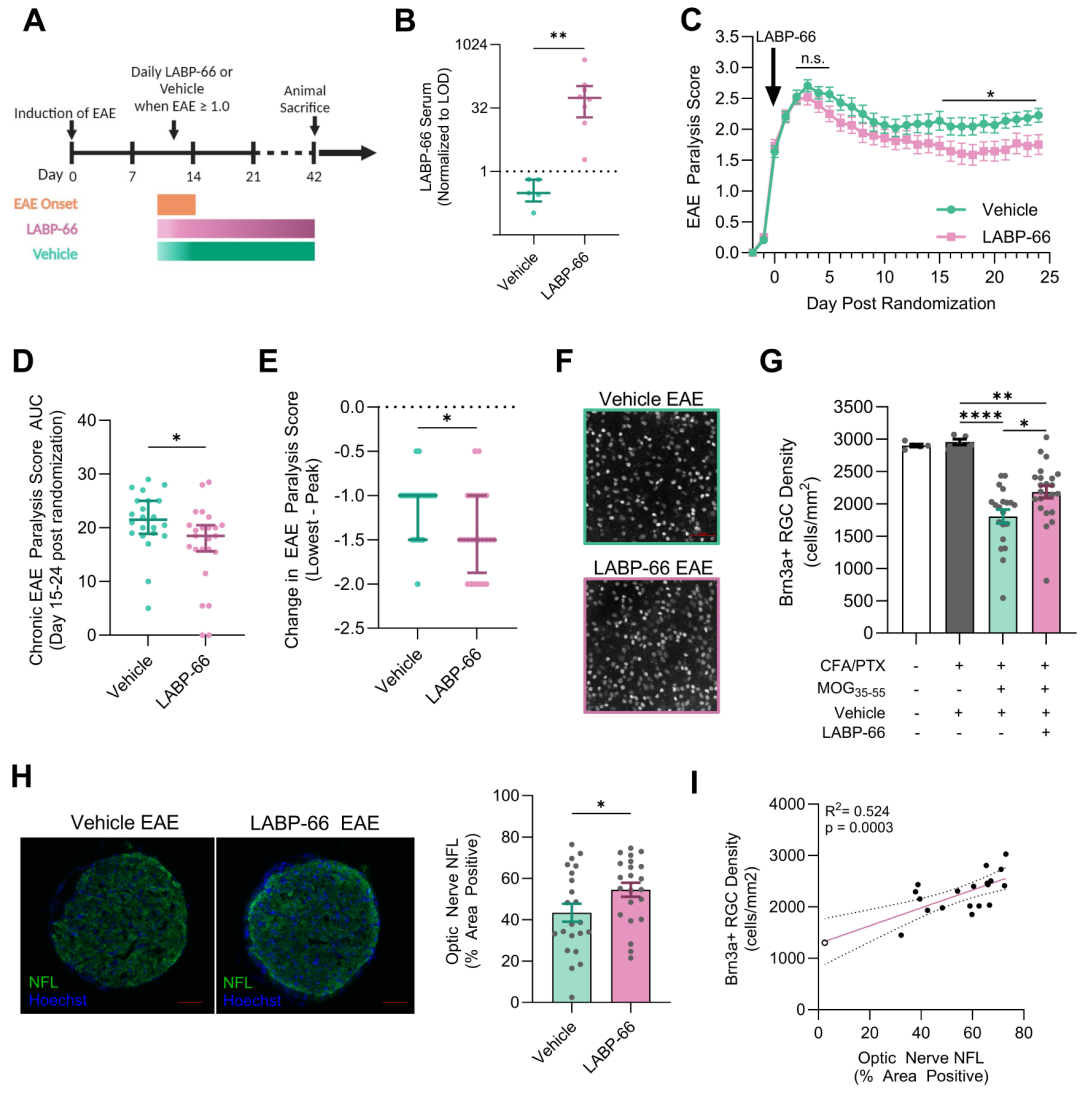
Paralysis severity and neurodegeneration in the anterior visual pathway in immunization EAE mice treated after paralysis onset with LABP-66. (**A**) Wild-type 12-week-old female mice were immunized against MOG_35 − 55_ to induce EAE. After the development of paralysis (EAE score 1.0 or greater), mice were randomized to daily oral gavage with LABP-66 (20 mg/kg; *n* = 22) or vehicle (0.5% methylcellulose; *n* = 24). At 42 days post-immunization mice were euthanized, and retina and optic nerves were isolated. (**B**) Serum concentration of LABP-66 2–4 h after either LABP-66 (20 mg/kg; *n* = 8) or vehicle (*n* = 5) oral gavage in WT EAE mice. The data was normalized to the assay limit of detection (LOD). Data presented as mean ± SEM with statistical analysis performed by Mann-Whitney U test. (**C**) EAE paralysis clinical score overtime normalized to day post randomization in LABP-66, and vehicle treated EAE mice with statistical analysis presented representing area under the curve analysis and acute and chronic time points. (**D**) EAE paralysis score area under the curve (AUC) analysis for days 15–24 post randomization. (**E**) Change in EAE paralysis score from peak score to lowest score post peak. Data for AUC and change in EAE score are presented as median ± interquartile range (IQR). (**F**) Representative IHC for Brn3a^+^ RGCs in the retina with (**G**) quantification of Brn3a^+^ RGC density vehicle- (*n* = 20) and LABP-66-treated (*n* = 23) EAE mice as well as naïve mice (*n* = 5 and CFA/PTX-only mice (*n* = 5). (**H**) Representative immunofluorescence (IF) images of neurofilament light chain (NFL) and average NFL percent area positive in the optic nerve of EAE mice treated with vehicle or LABP-66. (**I**) Correlation between percent area positive of NFL in the optic nerve to Brn3a^+^ RGC cell body density in the retina at day 42 post immunization in vehicle-treated and LABP-66 treated EAE mice, Correlation analysis was performed by Pearson correlation test. RGC density is presented as mean ± SEM with statistical analysis performed by one-way ANOVA with Tukey’s multiple comparison test. *p < 0.05, **p < 0.01, ***p < 0.001, ****p < 0.0001

To assess whether pharmacological activation of NLRX1 could ameliorate inflammatory demyelinating-associated neurodegeneration in vivo, we randomly assigned MOG_35 − 55_ immunized female mice, after the development of full tail paralysis (EAE score = 1), to receive daily gavage with either 20 mg/kg LABP-66 or vehicle (0.5% methylcellulose; Fig. 7A). We initiated LABP-66 treatment after the onset of paralysis to establish a post-treatment paradigm and avoid potential effects of LABP-66 on EAE induction and initial T-cell infiltration. LC-MS analysis (Eurofins Scientific) confirmed detectable absorption of LABP-66 in the serum of EAE mice treated with LABP-66 approximately 3 h after gavage (Fig. 7B). Successful randomization was confirmed by the absence of significant differences in timing post-induction of randomization, EAE paralysis score at randomization, or peak EAE paralysis score between LABP-66-treated and vehicle-treated mice (Suppl. Fig. 9A-C). Additionally, no significant differences in weight were observed between LABP-66-treated and vehicle-treated mice (Suppl. Fig. 9D). While there were no differences in early or peak EAE scores (Suppl Fig. 9E), LABP-66-treated mice exhibited lower average EAE scores at chronic time points (Fig. 7C–E). Specifically, LABP-66-treated mice had a significantly lower EAE score area under the curve at chronic time points, greater improvement from peak to lowest-post-peak EAE score, and a lower best post-peak EAE score (Suppl. Fig. 9F).

When evaluating retinal ganglion cell (RGC) density, we observed the expected loss of RGCs in vehicle-treated EAE mice compared to controls. However, LABP-66-treated EAE mice demonstrated significantly less RGC loss compared to vehicle-treated counterparts (Fig. 7F,G). Additionally, optic nerve neurofilament light chain (NFL) expression was significantly higher in LABP-66-treated mice compared to vehicle-treated mice (Fig. 7H). Consistent with prior experiments, there was a significant positive correlation between optic nerve NFL expression and RGC density in this cohort (Fig. 7I). We performed similar EAE experiments in *Nlrx1*^*−/−*^ mice and found that the therapeutic effect of LABP-66 is dependent on NLRX1. There was no improvement in clinical scores (Suppl. Fig. 9G) or RGC counts (Suppl. Fig. 9H) in LABP-66-treated *Nlrx1*^*−/−*^ EAE mice compared to vehicle-treated *Nlrx1*^*−/−*^ CFA controls. In the optic nerve, we quantified CD3+T cells, as well as Iba1 and GFAP expression, in LABP-66-treated EAE mice and found no significant changes compared to vehicle-treated EAE mice (Suppl. Fig. 10A-C). We quantified the effect of LABP-66 on immune infiltration and cytokine production by flow cytometry. We found that LABP-66 does not affect immune cell infiltration or the production of IFNγ and IL-17 cytokines by T cells. (Suppl. Fig. 10D–G). These results highlight the therapeutic potential of activating NLRX1 with LABP-66 in limiting neurodegeneration in the anterior visual pathway.

## Discussion

The continued prevalence of progressive neurodegenerative disease in many people living with MS, despite the effectiveness of disease-modifying therapies for relapses, strongly emphasizes the critical need for adjunctive therapies that protect neurons and control the persistent neuropathological processes within the CNS of these patients. Processes that may drive progressive disease include compartmentalized innate immune activation within the CNS, neurotoxic and oligotoxic reactive glia, CD8 + tissue-resident memory T-cells, and meningeal lymphoid aggregates, among others [65]. In our attempt to identify targets that may modulate these neurodegenerative processes within the CNS, we have demonstrated a neuroprotective role of the innate immune regulator NLRX1 within the anterior visual pathway in multiple EAE models. The anterior visual system is an ideal model to evaluate neurodegeneration in EAE because inflammation and demyelination in the optic nerve cause RGC cell-body neurodegeneration reliably and reproducibly in the retina. Using both immunization-based and spontaneous transgenic models, our research has demonstrated that the absence of NLRX1 leads to an exacerbation of RGC cellular stress and ultimately neurodegeneration in the retina and optic nerve. Notably, the neuroprotective role of NLRX1 is independent of both the severity of paralysis and the extent of immune infiltration into the CNS at the peak timepoint.

A previous study by Eitas et al. found that *Nlrx1*^*−/−*^ mice developed more severe classical EAE clinical symptoms compared to WT mice [20]. However, we have not observed a difference in clinical scores between WT and *Nlrx1*^*−/−*^ EAE mice in our vivarium, even when using the same immunization protocol. Remarkably, we also replicated our findings in other EAE models (OSE and adoptive transfer) and did not see alteration of the disease onset or course in EAE. One possible reason for this variability could be differences in environmental factors between vivariums, such as the gut microbiome and seasonality [19]. Despite having no impact on the clinical score or inflammatory infiltrate, the neuroprotective role of NLRX1 was significant. This suggests that the loss of NLRX1 affects neurodegenerative processes within the CNS, and that any role it may have in priming peripheral immune cells and trafficking into the CNS may be less critical.

While our spontaneous OSE model corroborated the protective role of NLRX1 in inflammatory neurodegeneration that we observed in our immunization EAE model, there were multiple unique and notable features of anterior pathway neurodegeneration in the OSE model. In our immunization EAE model, RGC neurodegeneration always accompanied clinical paralysis [28]. However, in our OSE model some mice had profound RGC loss despite no clinical evidence of paralysis and a few mice even showed little to no RGC loss despite development of significant clinical paralysis. These findings demonstrate greater pathologic independence between the development of optic neuritis and myelitis in the spontaneous OSE model as compared to immunization EAE. Interestingly, while TCR^MOG^ transgenic mice rarely spontaneously develop classical EAE/paralysis, a higher proportion do develop spontaneous optic neuritis over time [30, 66], which we have shown is associated with RGC loss. On the other hand, IgH^MOG^ transgenic mice never develop spontaneous paralysis, though they may develop subtle optic neuropathy [46, 67]. Taken together, our findings from both active and OSE models suggest that NLRX1 provides significant protection against RGC loss, driven by inflammatory optic neuritis [68].

We have previously shown that reactive glia at the peak of EAE are associated with RGC loss at the chronic phase of EAE [22, 28]. Our EAE data suggests that severe RGC loss in *Nlrx1*^*−/−*^ mice is not spontaneous but occurs only at the chronic stage of EAE. Additionally, twice as many RGCs in *Nlrx1*^−/−^ EAE mice RGCs are positive for phospho-c-Jun expression and have associated decreased Brn3a expression compared to WT EAE mice at acute time points prior to any observable RGC loss, consistent with increased neuronal cell stress and risk for subsequent degeneration. This increased RGC cellular stress in *Nlrx1*^−/−^ EAE mice may be the result of increased innate immune cytokines such as IL-1β and IL-6. Interestingly, IL-1β and inflammasome activation have been described in RGCs and retinal astrocytes in association with RGC loss in glaucoma models, demonstrating a link between innate immunity and neurodegeneration [69]. These data support that excessive CNS inflammation at the peak of EAE in *Nlrx1*^−/−^ mice contributes to the worsening of neuronal death at later stages, along with possible increased sensitivity of *Nlrx1*^*−/−*^ neurons to cell death.

To better understand the neuroprotective role of NLRX1 against inflammatory neurodegeneration in our models, we first aimed to investigate the how changes in NLRX1 expression correlate with inflammatory processes at different stages of the disease, particularly before the onset and during the peak of EAE. Our Western blot data show that NLRX1 protein levels remain unchanged during the preclinical and early clinical stages of EAE. However, at the peak of inflammation, NLRX1 levels began to decline in the spinal cord of EAE mice compared to CFA controls. Similar to our finding, a prior study reported reduced NLRX1 expression in a mouse model of inflammatory bowel disease during Dextran Sulfate Sodium (DSS) challenge [70]. In human patients, a significant downregulation of NLRX1 was observed in brain injury following aneurysm [19]. NLRX1 was originally identified as a brake on inflammation and innate immune activation [16, 17, 71], and its downregulation under inflammatory conditions may contribute to greater inflammation and disease progression.

The inflammatory state of EAE model has been extensively characterized in the literature [72, 73]. To investigate whether NLRX1 regulates inflammatory responses in the CNS during EAE, we quantified the expression of inflammatory mediators in the spinal cords of WT and *Nlrx1*^*−/−*^ EAE mice at the peak of disease. Since NLRX1 is known to regulate the canonical NF-κB pathway and type I interferon (IFN-I) responses [16, 17], we examined the expression of key inflammatory mediators associated with these pathways in the spinal cords of WT and *Nlrx1*^*−/−*^ EAE mice at the peak of disease. Consistent with previous studies, we found that NLRX1 selectively regulates certain NF-κB-driven genes including *Il6*,* Nos2*,* Il1b*,* Il1a*,* as well as Ccl2 and Cxcl1* chemokines, both of which are potent chemoattractant for myeloid cells. Our findings are consistent with previous studies, highlighting the role of NLRX1 in regulating major inflammatory pathways in the CNS [17, 22].

While we do not observe evidence of direct effect of *Nlrx1*^−/−^ or NLRX1 activators on the adaptive immune system in our EAE models, loss of NLRX1 has been shown to regulate the activation state of effector T-cells in other inflammatory models [18]. Thus, NLRX1 activity within T-cells could also downregulate the activation state of T-cells [74], thereby influencing the cascade of ongoing immune responses within the CNS. To exclude the potential effects of NLRX1 on T cell responses and to specifically investigate its role in innate immune cells, we established an adoptive transfer EAE model using lymphocyte-deficient recipient mice. We transferred highly active myelin-specific Th17 cells to *Rag*^*−/−*^ mice *and Nlrx1*^*−/−*^*Rag*^*−/−*^ mice to minimize the impact of NLRX1 deficiency on T cell priming and effector function during EAE. Following adoptive transfer, myelin-specific Th17 cells primarily infiltrate the brain and optic nerve, leading to extensive immune infiltration and acute inflammatory optic neuritis [75]. When we examined optic nerves in this model, we found that *Nlrx1*^*−/−*^*Rag*^*−/−*^ mice exhibited significantly increased GFAP and Iba1 expression at the peak timepoint compared to WT mice, indicating a severe glial response to inflammation and the potential regulatory effect of NLRX1 on glial activation in this model. We further examined the infiltration of T cells and myeloid cells to the optic nerve and brain of these mice and found no significant difference in the percentage of immune cells present in the optic nerve and brain parenchyma. Our findings suggest that NLRX1 deficiency enhances glial activation in this neuroinflammatory model. Compared to active EAE, glial activation has been shown to be more severe in the adoptive-transfer EAE [69]. This could potentially explain why we did not observe significant differences in the expression of these markers during active EAE, as the less severe glial activation in the active EAE model may have masked any subtle changes related to NLRX1 deficiency.

Since we found that NLRX1 regulates innate immune inflammatory processes at the peak of EAE, we sought to investigate this inflammatory signature in astrocytes, the most abundant glial cells in the CNS, which play a significant role in neurodegeneration, particularly at the chronic stage. We evaluated the transcriptome in LPS/IFNγ activated WT and *Nlrx1*^*−/−*^ astrocytes using primary astrocyte culture and found the dysregulation of key molecular pathways associated with neurodegeneration, including NF-κB signaling, oxidative phosphorylation, unfolded protein response, and the E2F1 pathway.

The canonical NF-κB signaling pathway is one important innate immune activation signaling pathway that is highly activated in MS disease-associated astrocytes and microglia in the brain parenchyma, particularly beneath meningeal lymphoid aggregates; at the lesion edge of slowly expanding lesions; and in the optic nerves in MS, as well as in EAE [7–10, 68]. Pathologic TLR4/NF-κB activation in microglia and astrocytes can result in neurotoxic and oligotoxic microglia [76, 77]. Ponath et al. demonstrated that a known MS-risk variant (rs7665090^G^) located near *NFKB1* increased NF-κB signaling in MS lesions and associated with decreased astrocyte glutamate uptake and lactate release in vitro, highlighting direct detrimental effects on astrocyte homeostatic functions important for neuronal health [78]. In line with these findings, a prior study demonstrated that transgenic inhibition of NF-κB in GFAP + astroglial decreased optic neuritis and prevented RGC loss in active MOG_35 − 55_ EAE [68]. Consistent with our RNAseq data, NLRX1 has been shown to negatively regulate NF-κB signaling through targeting of TRAF6 and IKK [16, 17]. Homeostatic dampening of NF-κB activation through pharmacologic targeting of NLRX1 may be able to convert chronically active, neurotoxic, and oligotoxic microglia and astrocytes back to a more homeostatic or physiologic state.

NLRX1 has also been shown to enhance glutamate uptake and inhibit glutamate release from astrocytes, possibly through its ability to enhance mitochondrial ATP production [79]. Consistent with these findings, we have demonstrated that *Nlrx1*^*−/−*^ astrocytes in response to LPS/IFNγ stimulation have decreased relative induction of oxidative phosphorylation genes, including multiple subunits of all complexes (I-V) in the mitochondrial respiratory chain and glutamate dehydrogenase, compared to WT astrocytes. Interestingly multiple studies suggest that NLRX1 promotes oxidative phosphorylation in immune cells but downregulates oxidative phosphorylation and promotes aerobic glycolysis in cancer and non-immune cells [80], suggesting in this context astrocytes behave more like immune cells.

Our RNA sequencing data also identified that *Nlrx1*^*−/−*^ astrocytes compared to WT astrocytes have enhanced induction of unfolded protein response genes and a greater reduction in cell-cycle related targets of E2F transcription factors in response to LPS/IFNγ stimulation. These and several other NLRX1 cell-type dependent functions including roles in reactive oxygen species production, RNA processing, autophagy, and apoptosis may also contribute to the neuroprotective role of NLRX1 innate immune activation and reactive glia [81].

It is also possible that NLRX1 could play a cell-autonomous role in RGCs during neuroinflammation in vivo. Previous studies have demonstrated the neuroprotective role of NLRX1 in neuronal cell lines in vitro using a mitochondria stressor [82] or following traumatic brain Injury (TBI) in vivo [19]. NLRX1 role in regulating cell death and autophagy have been previously reported [83, 84]. NLRX1 also plays a key role in maintaining oxidative phosphorylation and cell integrity, while its loss increases oxygen consumption, oxidative stress, and apoptosis during ischemia-reperfusion injury [21]. These mechanisms could potentially contribute to the neuroprotective activity of NLRX1 against inflammatory neurodegeneration and merit further investigation.

Given the multifunctional properties of NLRX1 as both an immune regulator and a key modulator of cell survival pathways, its role extends beyond inflammatory responses to include the maintenance of cellular homeostasis and protection against neurodegeneration. We tested the potential therapeutic targeting of NLRX1 in our active EAE model and found that the novel NLRX1 activator LAPB-66 is neuroprotective against anterior pathway neurodegeneration, demonstrating for the first time neuroprotective effects of a pharmacologic NLRX1 activator in vivo. We further showed that these effects of LABP-66 are dependent on NLRX1, as we did not observe protection against RGC loss in *Nlrx1*^−/−^ EAE mice. Our in vitro studies revealed that LAPB-66 treatment limits the expression of NF-κB-regulated inflammatory gene in WT glia, while no change was found in *Nlrx1*^*−/−*^ glia, suggesting that the observed neuroprotective effects of LAPB-66 may be mediated through its anti-inflammatory activity, and is specific to NLRX1.

We have thus identified NLRX1 as a potential therapeutic target for inflammatory neurodegeneration mediated by the CNS innate immune activation. Due to the complexity of studying the functional outcomes of these pathways in global NLRX1 knockout mice, conditional *Nlrx1*^*−/−*^mice will be needed to further explore the role of NLRX1 in specific cell types in vivo.

## Conclusion

Our studies highlight the potential of targeting NLRX1 as a neuroprotective strategy against inflammatory neurodegeneration, particularly through its immunomodulatory effects outside of the adaptive immune system. These results indicate that the observed axonal and synaptic deficits are not driven by increased immune cell infiltration but are more likely due to intrinsic neuronal vulnerabilities or glial inflammatory mechanisms exacerbated by the absence of NLRX1. As a negative regulator of NF-κB-activated inflammatory glia, NLRX1 may help reduce the activity of neurotoxic and oligotoxic glia in chronic neurodegenerative diseases, such as MS, Alzheimer’s, and Parkinson’s disease, where growing evidence suggests CNS-compartmentalized inflammation and chronic-reactive glia are significant contributors. This research provides the first evidence that targeting the NLRX1 pathway with small molecule activators is neuroprotective and could be a promising therapeutic target for limiting chronic innate immune activation in inflammatory neurodegenerative conditions such as progressive multiple sclerosis, which currently lack effective treatments. Our studies support continued research of NLRX1 and NLRX1-targeted strategies for chronic neuroinflammatory and neurodegenerative disorders. 

## Electronic supplementary material

Below is the link to the electronic supplementary material.

Supplementary Material 1

Supplementary Material 2

Supplementary Material 3

## Data Availability

Raw sequencing data, as well as transcript level quantifications generated by Salmon and normalized counts per million generated by limma-voom, for the RNAseq experiment, is available at the Gene Expression Omnibus with the accession GSE270482 (Accession is private and will be made public prior to publication, reviewer access code yhsjwsuqztsflwx).

## Abbreviations

CFA: Complete Freund’s adjuvant
CNS: Central nervous system
EAE: Experimental autoimmune encephalomyelitis
MS: Multiple sclerosis
NF-κB: Nuclear factor kB
NFL: Neurofilament light chain
NLRX1: Nucleotide binding and oligomeric domain-like receptor X1
OCT: Optical Coherence Tomography
PMS: Progressive MS
Rag: Recombination-activating gene
RGC: Retinal ganglion cell
RRMS: Relapsing remitting MS
TCR: T cell receptor
TLR: Toll-like receptor
WT: Wild type
