# Supplementary material for: NLRX1 limits inflammatory neurodegeneration in the anterior visual pathway
Source: J Neuroinflammation. 2025 Jan 28;22:21. doi: 10.1186/s12974-025-03339-0 (PMC11773851; doi:10.1186/s12974-025-03339-0)
Supplement: Supplementary file 1 — Supplementary Material 1 [file 12974_2025_3339_MOESM1_ESM.docx]

**Supplementary Figures and Legends**


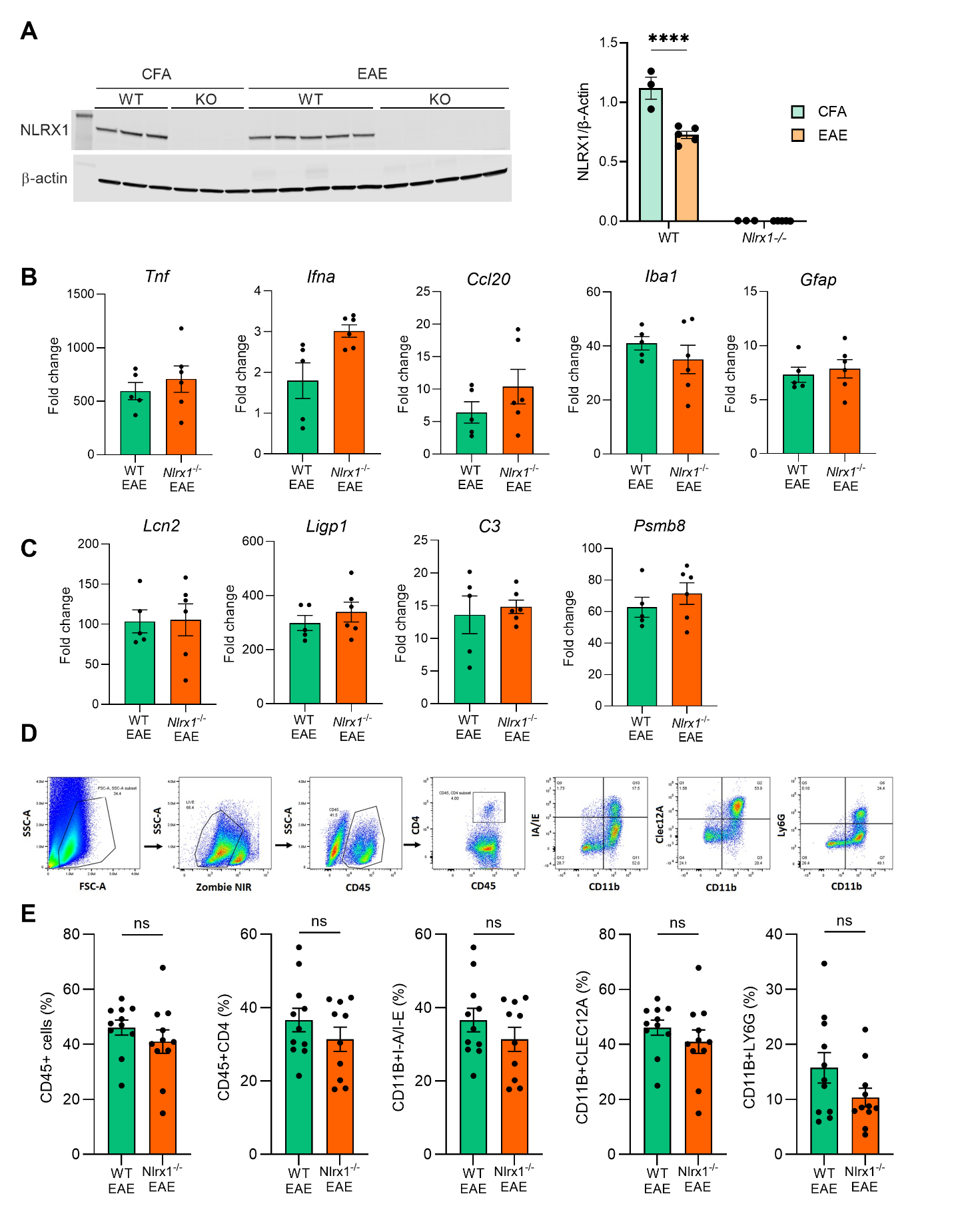


**Supplementary Figure 1. Spinal cord inflammation at the peak of acute EAE.**

**(A)** NLRX1 protein expression by Western blot in the spinal cord of WT and *Nlrx1^-/-^* mice CFA-treated (n=3 per genotype) and EAE mice (n=5 per genotype). Gene expression analysis of proinflammatory mediators **(B)** and reactive astrocytes associated genes **(C)** in the spinal cord of WT (n=5) and *Nlrx1^-/-^* (n=6) mice at PID14. Gene expression was normalized CFA-treated mice (data not shown). **(D)** Gating strategy and **(E)** the quantification of immune infiltrate to the spinal cord at the peak EAE (PID14), including CD45^+^ cells as a proportion of total live cells; CD45^+^CD4^+^ cells, CD11b^+^Clec12A^+^ cells, CD11b^+^LY6G^+^ cells, and CD11b^+^I-A/I-E^+^ cells as a proportion of total CD45^+^ live cells. Data presented as mean ± SEM. Western blot data was compared using 2-way ANOVA with Tukey’s multiple comparison test. Gene expression data was analyzed using Fisher’s LSD test. Flow cytometry data was analyzed by Student’s t-test. ****p<0.0001.


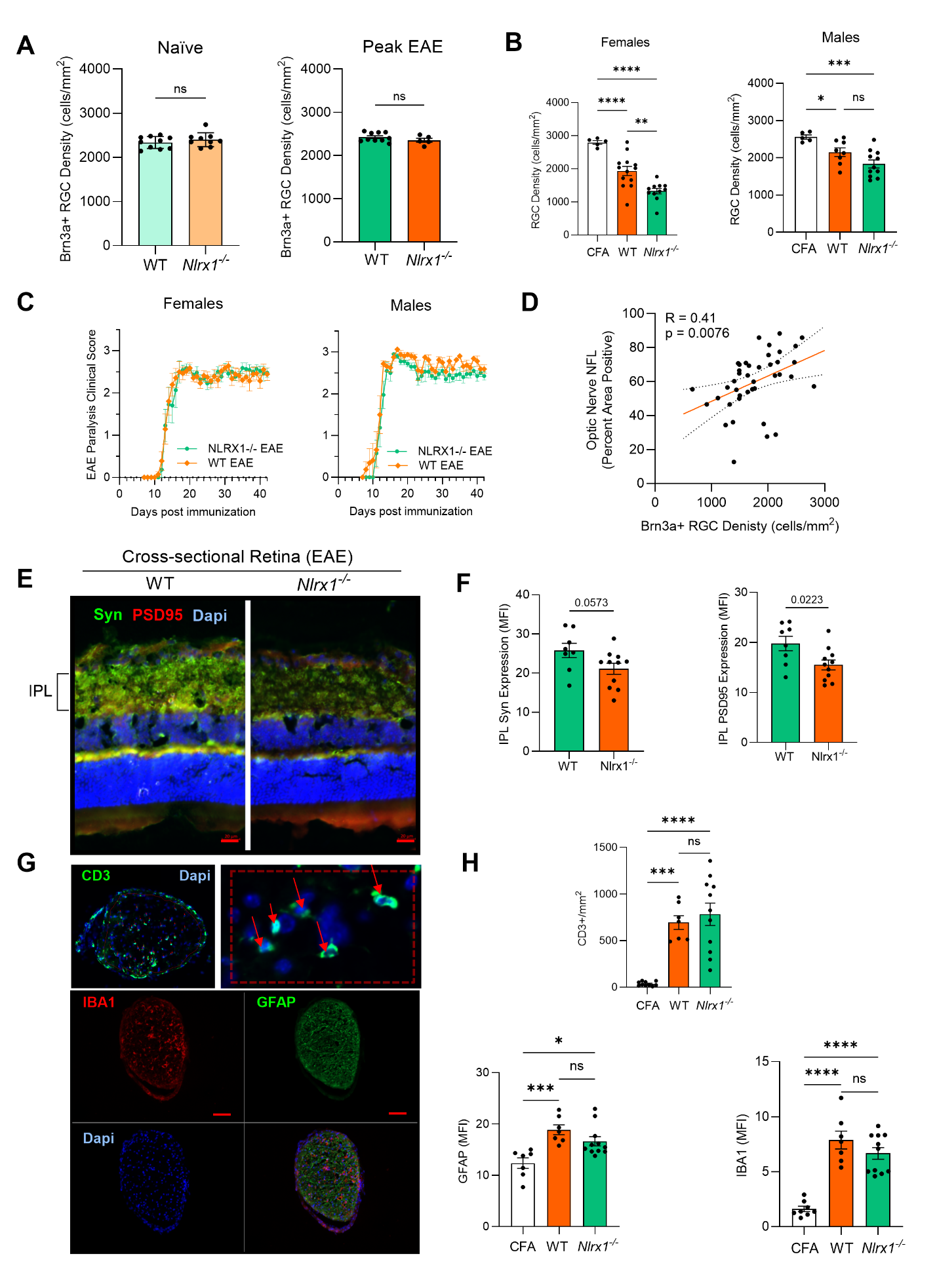


**Supplementary Figure 2. Anterior visual pathway degeneration, outcomes by sex, and optic nerve immune cell infiltration and glial activation at the chronic timepoint in WT and *Nlrx1*^-/-^ active EAE mice. (A)** RGC density in flat-mounted retinas from naïve healthy WT and *Nlrx1^-/-^* mice at 10-week-old and EAE mice at the peak timepoint (PID14). **(B)** Quantification of RGC counts in flat-mounted retinas from WT and *Nlrx1^-/-^* EAE mice at PID 42, stratified by sex. **(C)** Active EAE behavioral score in WT and *Nlrx1^-/-^* mice, analyzed separately by sex. **(D)** Correlation between RGC cell body density in the retina and percent and NFL percent area positive in the optic nerve in WT and *Nlrx1^-/-^* immunization EAE mice (n=41) at PID 42. Statistical analysis performed by Pearson correlation. **(E)** Representative IF staining of cross-sectional retina for PSD95 and alpha-synuclein (Syn) in WT and *Nlrx1^-/-^* immunization EAE mice at PID 42. **(F)** Average MFI of Synaptophysin and PSD95 in the inner plexiform layer in WT (n=8) and *Nlrx1^-/-^* (n=11) immunization EAE mice at PID 42. **(G)** Optic nerves were isolated from MOG_35-55_ immunization EAE mice 42 days after immunization. Representative images of immunofluorescence staining and **(H)** the quantification of CD3+ T-cell density, Iba1 MFI, and GFAP MFI in CFA-controls (n=7-10), WT (n=7) and *Nlrx1^-/-^* (n=11) EAE mice. Data presented as mean at ± SEM with statistical analysis by Student’s t-test. *p<0.05.


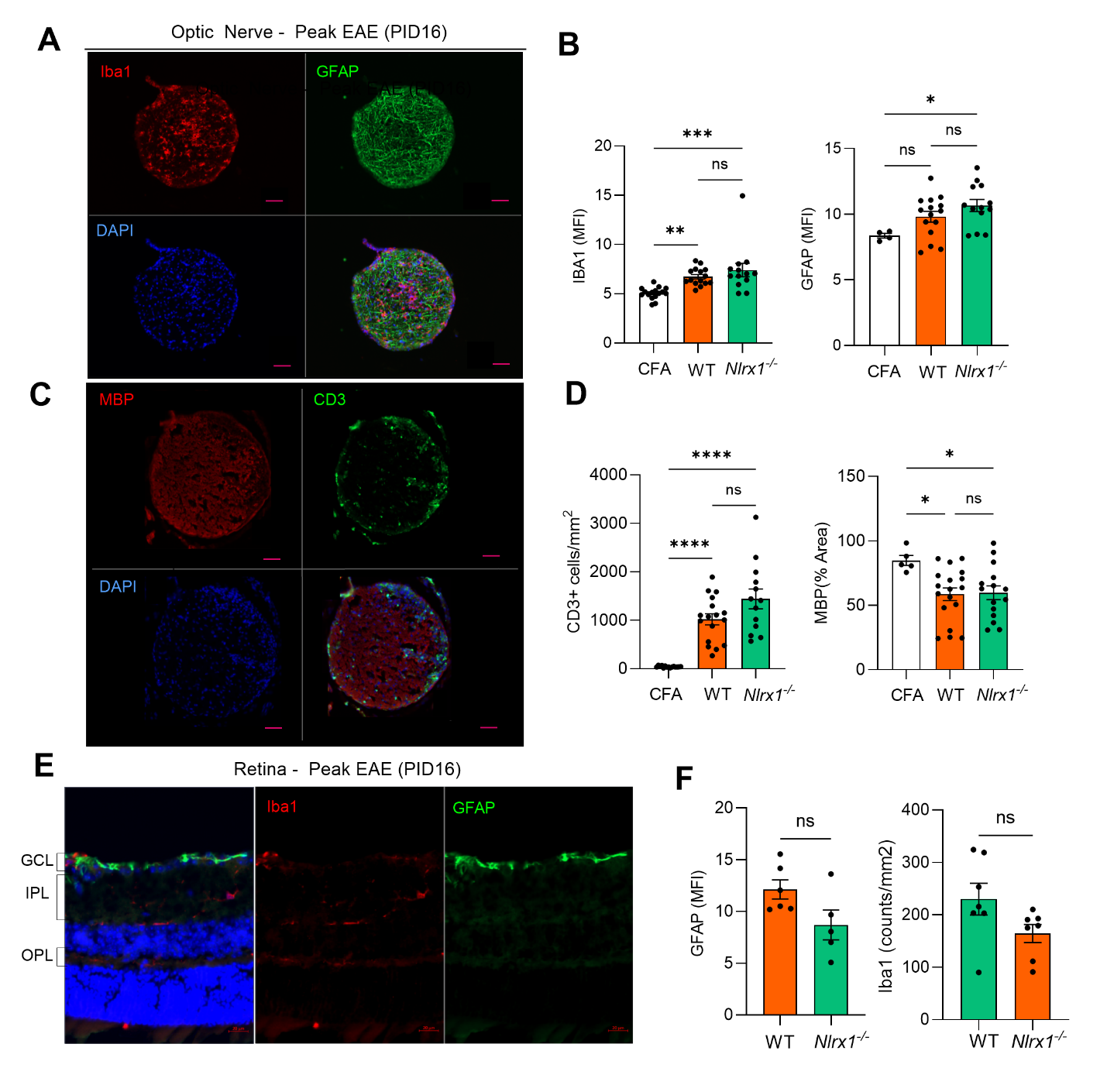


**Supplementary Figure 3. Optic nerve and retina immune cell infiltration and glial activation at the peak timepoint in WT and *Nlrx1^-/-^* active EAE mice. (A)** Optic nerves were isolated from MOG_35-55_ EAE mice 16 days after immunization (PID16). Proximal, medial, and distal segments of the optic nerve were analyzed by immunofluorescence staining. **(A)** Representative images of optic nerves stained with antibodies against Iba1 and GFAP **(B)** and the quantification of mean fluorescent intensity (MFI) in CFA-only (n=4-16), WT (n=15-17), and *Nlrx1^-/-^* (n=13) EAE mice 16 days after immunization. **(C)** Representative images of DAPI, CD3, and MBP with **(D)** quantification of CD3+ T-cell density and percent area positive of MBP. **(E)** Representative images of retina stained with anti-Iba1 and anti-GFAP antibodies and **(F)** the quantification of GFAP mean fluorescent intensity (MFI) in the ganglion cell layer (GCL) and Iba1 counts in the inner and outer plexiform layers (IPL, OPL) in WT (n=7), and *Nlrx1^-/-^* (n=7) EAE mice 16 days after immunization. Representative images were taken at 20x magnification, scale bar =20µM. All average data are presented as mean ± SEM with statistical analysis performed by one-way ANOVA with Tukey’s multiple comparison test. *p<0.05, **p<0.01, ***p<0.001, ****p<0.0001.


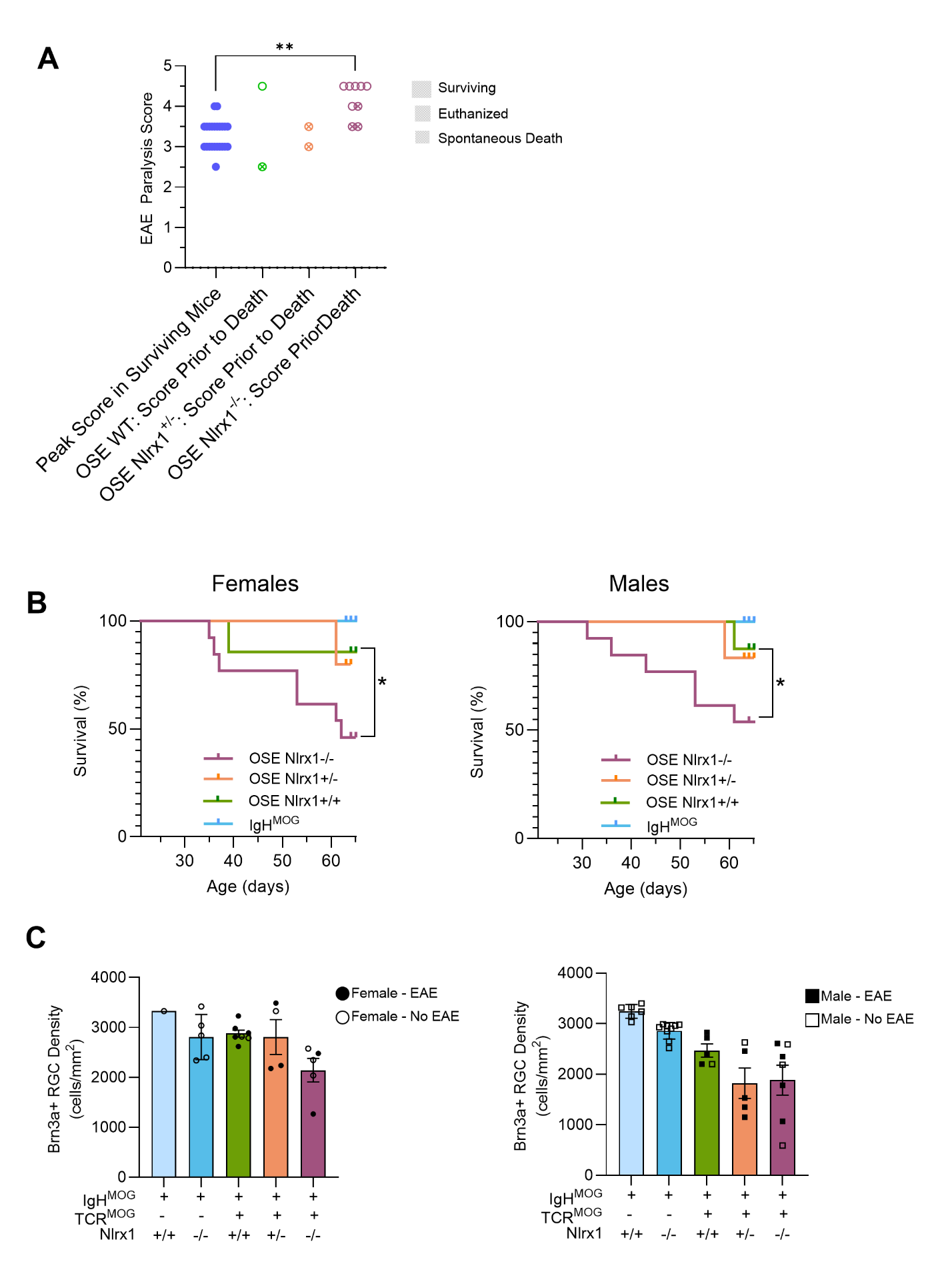


**Supplementary Figure 4. Survival and RGC counts by sex in *Nlrx1*^+/+^, *Nlrx1*^+/-^, and *Nlrx1*^-/-^ OSE mice. (A)** EAE clinical paralysis severity in *Nlrx1^-/-^* opticospinal encephalomyelitis (OSE) mice. We analyzed EAE clinical paralysis score prior to spontaneous death or euthanasia (score of 4.5 or severe 4.0) in WT, *Nlrx1^+/-^,* and *Nlrx1^-/-^* OSE mice and peak EAE score of all surviving OSE mice. Statistical testing performed by Kruskal-Walls H-test with post-hoc Dunn’s multiple comparison test. **(B)** Survival plots and **(C)** Brn3^+^ RGC density of surviving *Nlrx1***^+/+^** , *Nlrx1^+/-^*, and *Nlrx1^-/-^* OSE mice, stratified by sex. Square and circle data points identify male and female mice, respectively. Filled in and open data points identify mice that developed clinical EAE and those that did not, respectively. Averages are presented as mean ± SEM with statistical analysis performed by one-way ANOVA with post hoc Dunnett’s multiple comparison test. *p<0.05, **p<0.01, ***p<0.001.


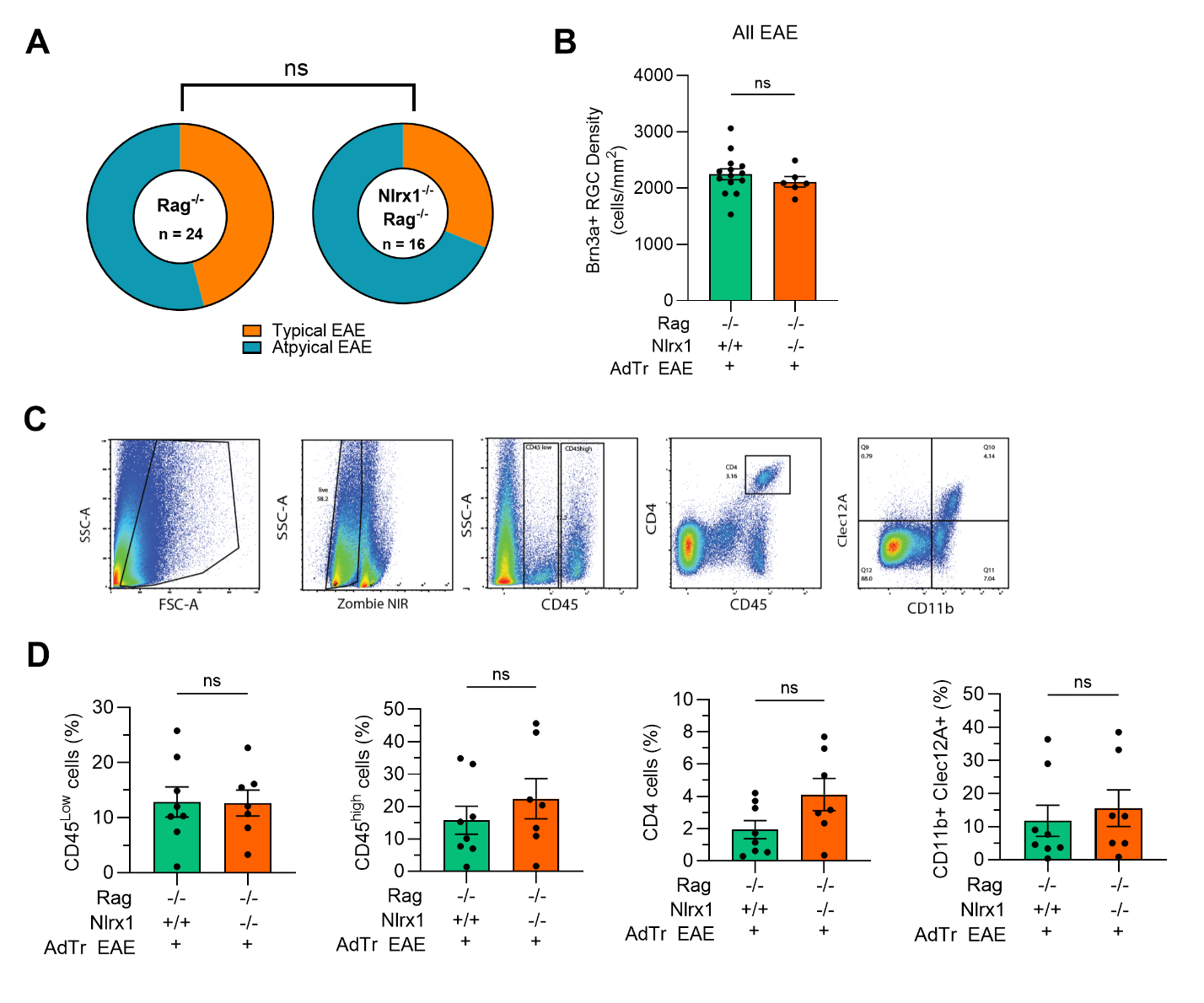


**Supplementary Figure 5: EAE clinical type, RGC density, and immune cell infiltration in adoptive transfer EAE *Rag^-/-^* and *Nlrx1^-/-^Rag^-/-^* mice. (A)** Ratio of typical EAE (ascending paralysis) or atypical EAE (ataxia and tremors) as initial clinical symptom onset in adoptive transfer *Rag^-/-^* and *Nlrx1^-/-^Rag^-/-^* mice. Statistical analysis comparing proportions of EAE type were performed by Fisher’s exact test. **(B)** Average RGC density of adoptive transfer *Rag^-/-^* and *Nlrx1^-/-^Rag^-/-^* mice at two weeks post injection of activated anti-MOG CD4^+^T-cells. **(C)** Gating strategy and flowcytometric analysis of immune cell infiltration to the brain of *Rag^-/-^* and *Nlrx1^-/-^Rag^-/-^* mice two weeks post adoptive transfer of activated CD4^+^T-cells. **(D)** The quantification of live immune cells including CD45^+^ cells (CD45 low and CD45 high populations), CD45^+^CD4^+^ cells, and CD11b^+^Clec12A^+^ cells as a proportion of total CD45^+^ live cells. Data depicting averages are presented as mean ± SEM with statistical analysis performed by Student’s t-test.


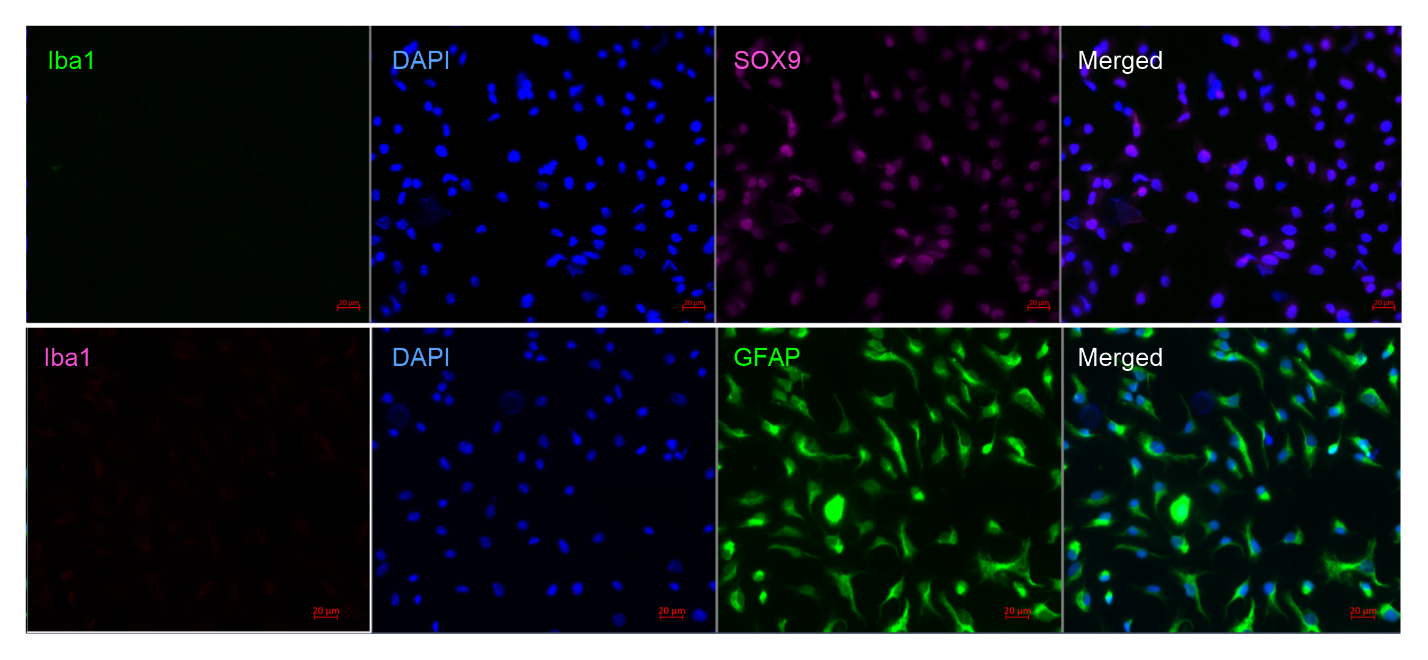


**Supplementary Figure 6. Expression of astrocyte and microglial markers in primary astrocyte cultures.** The purity of primary astrocyte cultures was assessed by immunofluorescence staining of cells. Representative images of immunofluorescence staining for the astrocyte markers GFAP and SOX9, the microglia marker Iba1, and the nuclei marker DAPI in primary astrocyte cultures. No Iba1 staining was detected with two different Iba1 antibodies.


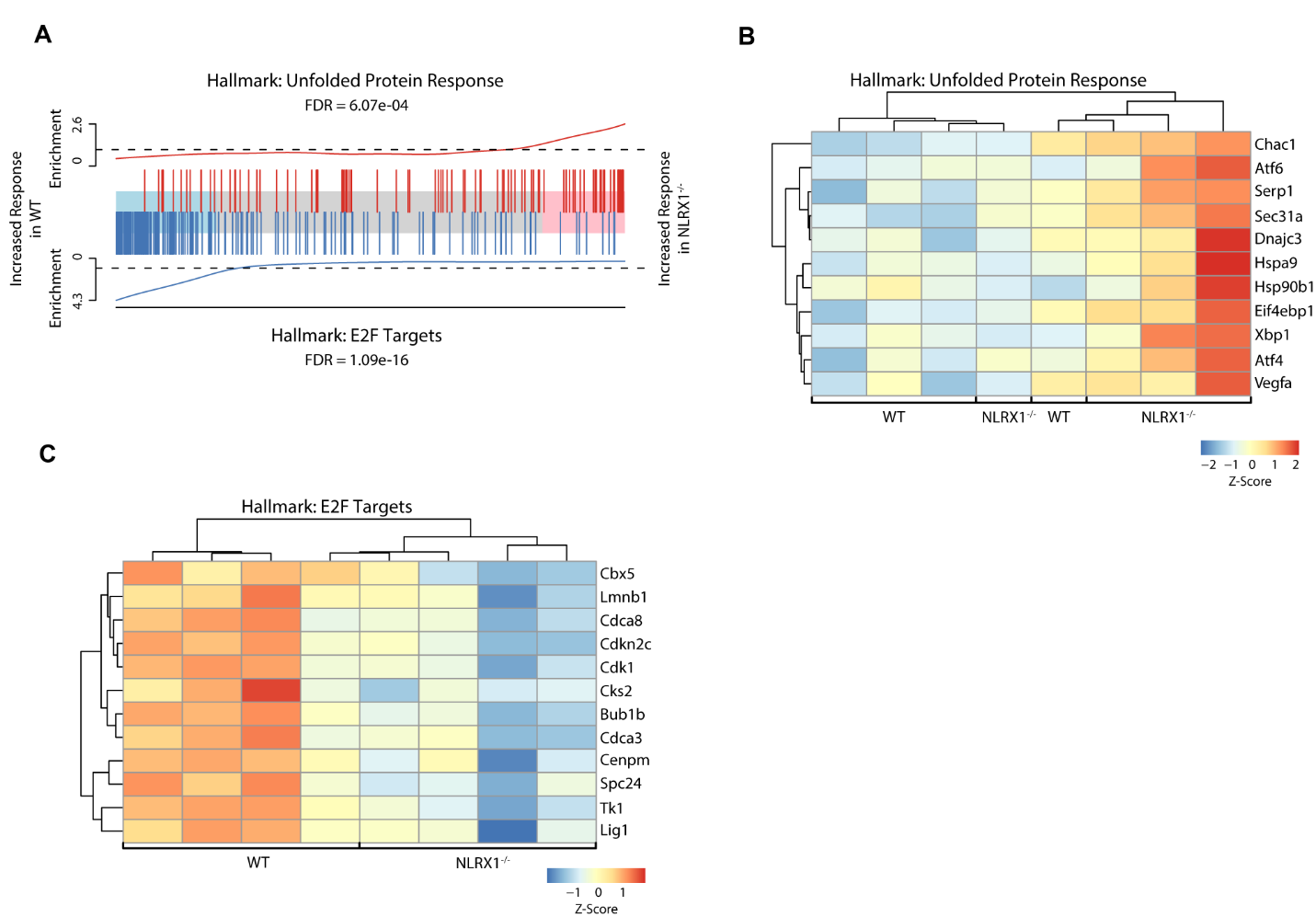


**Supplementary Figure 7*.* Transcriptome in LPS +IFNγ-stimulated WT and *Nlrx1*^-/-^ primary astrocytes.** Extension of Fig. 5. **(A)** Barcode plot showing genes associated with the Unfolded Protein Response (top, red) and E2F targets (bottom, blue) ranked from left to right where genes on the left were upregulated more in WT than *Nlrx1*^-/-^ in response to LPS+IFNγ while genes on the right were upregulated more in *Nlrx1*^-/-^ than in WT. Heatmaps showing normalized log 2-fold change values (LPS+IFNγ stimulated vs unstimulated/vehicle) for a selection of genes associated with **(B)** Unfolded Protein Response and **(C)** E2F targets where each row represents a gene and each column represents an independent biological replicate.


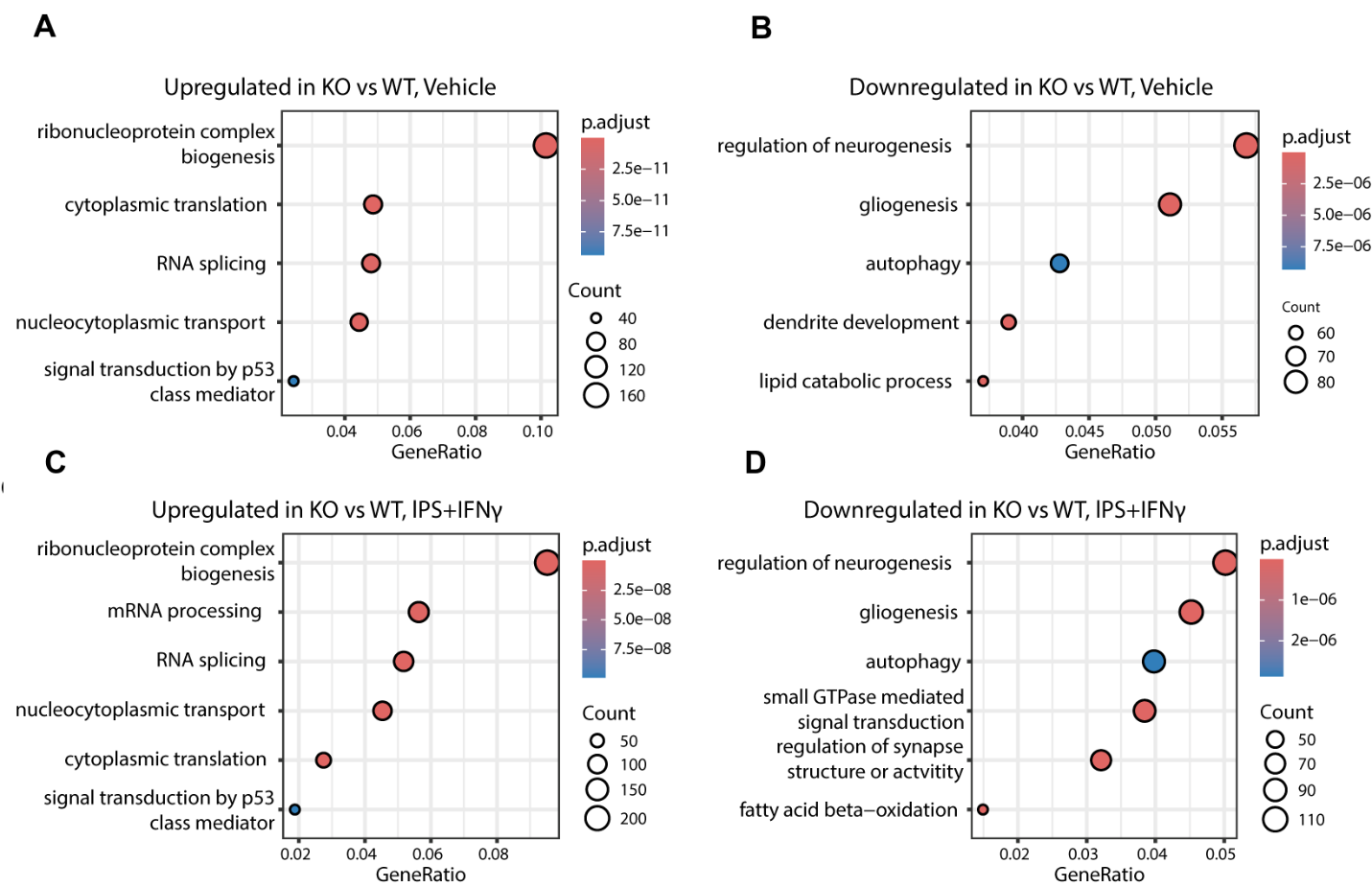


**Supplementary Figure 8. Transcriptome in unstimulated and LPS+IFNγ-stimulated WT and *Nlrx1^-/-^* primary astrocytes. (A-D)** Dotplot showing results of hypergeometric testing of gene ontology (GO) term enrichment in the genes upregulated (**A, C)** or downregulated (**B, D)** in *Nlrx1*^-/-^ vs WT in either vehicle alone (**A, B)** or in LPS+IFNγ alone (**C, D)**. X-axis represents the gene ratio which is the number of genes associated with the GO term that were differentially expressed divided by the number of genes differentially expressed. Dot size represents how many genes associated with that term were differentially expressed. Dot color represents p-value adjusted for false discovery rate. Pathways shown are a selection of the top pathways in each condition and direction. See Supplementary Tables 1-8 for full results of differential expression and GO term enrichment.


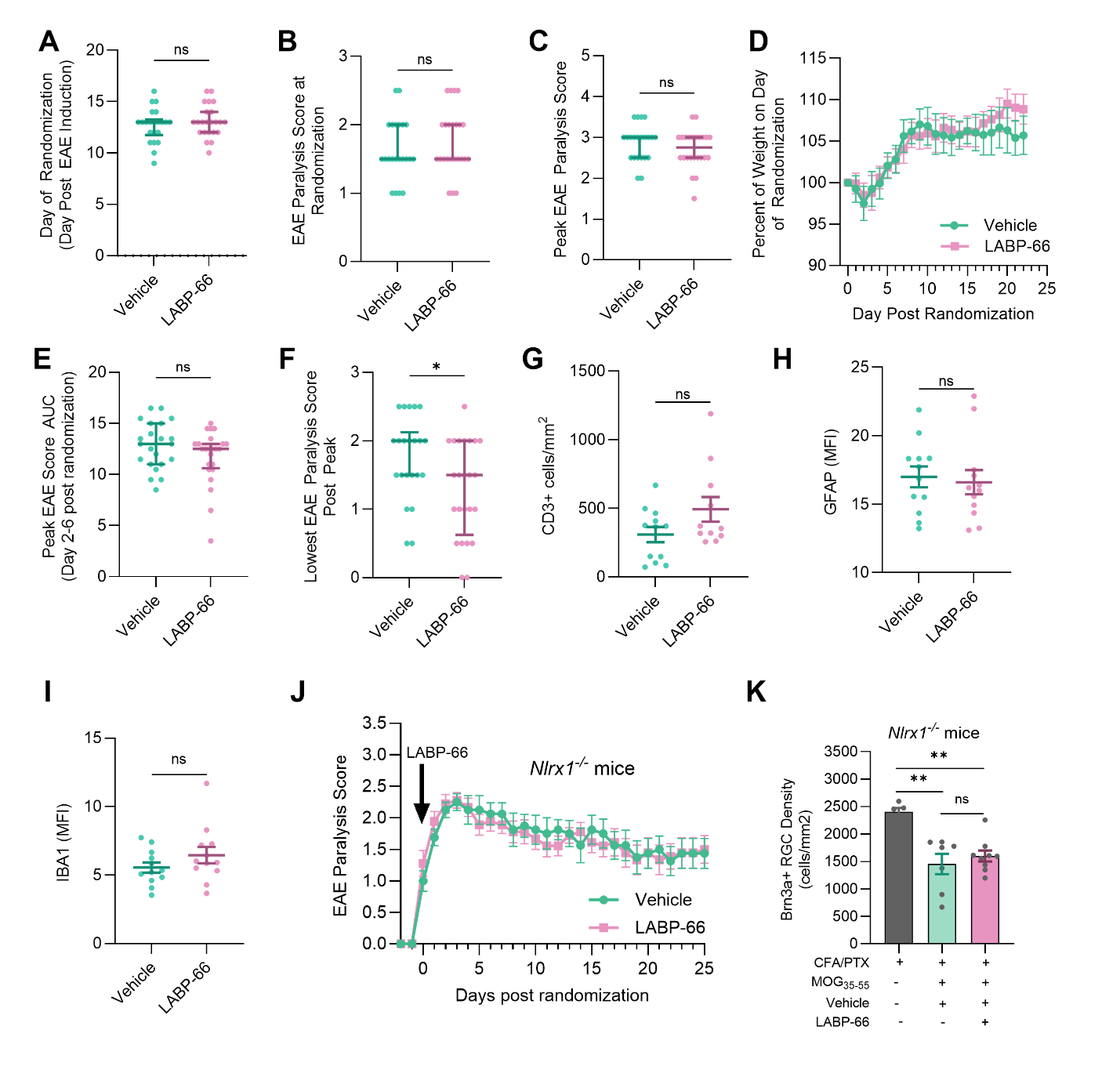


**Supplementary Figure 9: Randomization, paralysis severity, and neurodegeneration in the anterior visual pathway in immunization EAE mice treated after paralysis onset with LABP-66.** Wild-type 12-week-old female mice were immunized against MOG_35-55_ to induce EAE. After the development of paralysis (EAE score 1.0 or greater), mice were randomized to daily oral gavage with LABP-66 (20 mg/kg) or vehicle (0.5% methylcellulose). At 42 days post-immunization mice were euthanized, and retina and optic nerves were isolated. Randomization was effective with no significant difference in **(A)** day of randomization (or day EAE paralysis clinical score ≥ 1.0), **(B)** EAE score at time of randomization, or **(C)** peak EAE paralysis score between vehicle-treated and LABP-66 treated EAE mice. **(D)** Percent change in weight from starting weight on day of randomization over time in vehicle-treated and LABP-66 treated EAE mice. **(E)** EAE paralysis score area under the curve (AUC) for days 2-6 post-randomization (peak EAE) and **(F)** lowest EAE paralysis score obtained post EAE peak. Quantification of **(G)** CD3^+^ T-cells, **(H)** GFAP MFI, **(I)** and Iba1 MFI in the optic nerves at day 42 post immunization in vehicle-treated and LABP-66 treated EAE mice by IF. **(J)** EAE paralysis clinical score overtime normalized to day post randomization in LABP-66 and vehicle-treated *Nlrx1^-/-^* EAE mice, and **(K)** quantification of Brn3a^+^ RGC density in the retina from vehicle- (n=8) and LABP-66-treated (n=9) *Nlrx1^-/-^* EAE mice. All data was presented as mean ± SEM with statistical analysis performed by one-way ANOVA with Tukey’s multiple comparison test or unpaired Student’s t-test. *p<0.05, **p<0.01.


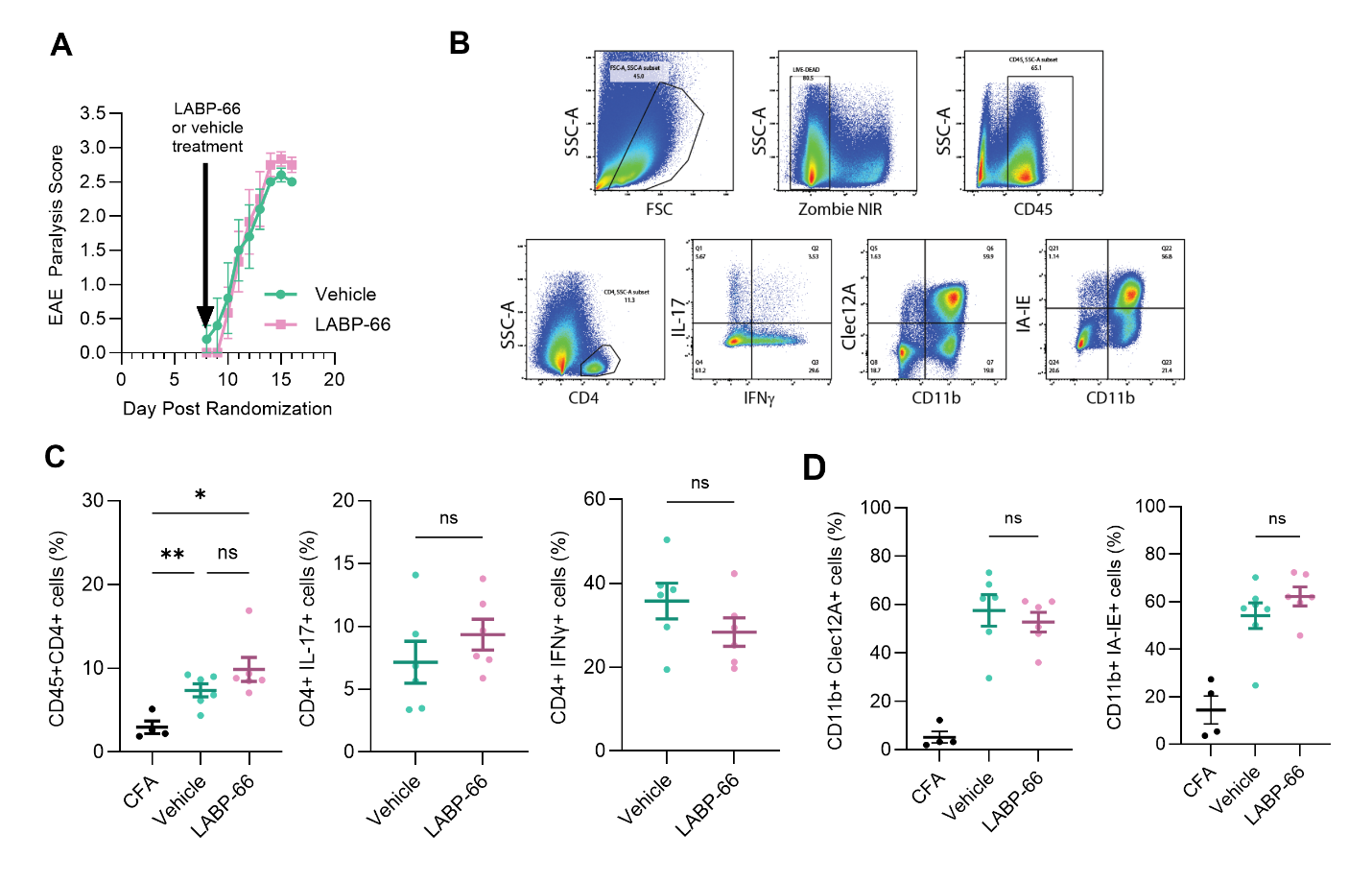


**Supplementary Figure 10. Effect of LABP-66 treatment on the immune cell infiltration into the spinal cord of mice at the peak of EAE.** **(A)** Wild-type 12-week-old female mice were immunized against MOG_35-55_ to induce EAE. After the development of paralysis (EAE score 1.0 or greater), mice were randomized to daily oral gavage with LABP-66 (20 mg/kg) or vehicle (0.5% methylcellulose). At 16 days post-immunization, mice were sacrificed, and spinal cords were isolated and processed for flow cytometry. **(B)** Gating strategy and **(C)** the quantification of immune infiltrate to the spinal cord. Cells including CD45^+^ cells as a proportion of total live cells; CD45^+^CD4^+^ cells, and the percentage of CD4+ cells that produce IFNγ or IL-17; **(D)** The percentage of myeloid cells, CD11b^+^Clec12A^+^ cells and CD11b^+^I-A/I-E^+^ cells as a proportion of total CD45^+^ live cells. All data is presented as mean ± SEM with statistical analysis performed by one-way ANOVA with Tukey’s multiple comparison test or unpaired Student’s t-test. *p<0.05, **p<0.01.
