# Supplementary material for: NLRX1 limits inflammatory neurodegeneration in the anterior visual pathway
Source: J Neuroinflammation. 2025 Jan 28;22:21. doi: 10.1186/s12974-025-03339-0 (PMC11773851; doi:10.1186/s12974-025-03339-0)
Supplement: Supplementary file 2 — Supplementary Material 2 [file 12974_2025_3339_MOESM2_ESM.zip › Supplementary Tables and Legends.docx]

**Supplementary Table 1: Differentially Expressed Genes – Interaction of Genotype and Stimulus**.

From bulk RNA-seq experiment comparing *Nlrx1^-/-^* and WT primary cultured astrocytes stimulated with and without LPS+IFNγ, gene list generated by DESeq2 testing for a differential effect of LPS+IFNγ on *Nlrx1^-/-^* astrocytes compared to WT astrocytes. baseMean: Mean normalized expression across all conditions. Log2FoldChange: Fold change comparing *Nlrx1^-/-^* to WT on a log 2-fold change scale. lfcSE: Standard error of Log2FoldChange. Stat: Test statistic calculated by DESeq2. Padj: P.value for gene being differentially affected by LPS+IFNγ in *Nlrx1^-/-^* vs WT after adjusting for multiple comparisons.

**Supplementary Table 2: CAMERA Analysis of Genotype Treatment Interaction**.

From bulk RNA-seq experiment comparing *Nlrx1^-/-^* and WT primary cultured astrocytes stimulated with and without LPS+IFNγ, results of competitive gene set test (CAMERA) analysis for enrichment of genes after testing for differential effect of LPS+IFNγ on *Nlrx1^-/-^* vs WT astrocytes using the MSigDb Mouse Orthologue Hallmark Pathways. NGenes: Number of genes in the pathway. Direction: Whether pathway is up (in *Nlrx1^-/-^*) or down (ie up in WT). FDR: PValue of enrichment adjusted for false discovery rate.

**Supplementary Table 3: Differentially Expressed Genes – *Nlrx1^-/-^* vs WT in Vehicle**

As in Supplementary Table 1. but testing for differentially expressed genes comparing *Nlrx1^-/-^* Vehicle condition to WT vehicle condition.

**Supplementary Table 4: Differentially Expressed Genes - *Nlrx1^-/-^* vs WT in LPS+IFNγ**

As in Supplementary Table 1, but testing for differentially expressed genes comparing *Nlrx1^-/-^* LPS+IFNγ condition to WT LPS+IFNγ condition.

**Supplementary Table 5: Upregulated GO terms in *Nlrx1^-/-^* vs WT in Vehicle**

From bulk RNA-seq experiment comparing *Nlrx1^-/-^* and WT primary cultured astrocytes stimulated with and without LPS+IFNγ, results of hypergeometric testing for enrichment of Biological Processes Gene Ontology Terms in the genes upregulated in *Nlrx1^-/-^* vs WT in Vehicle. GeneRatio: Ratio of genes differentially expressed and associated with the GO term to the total number of genes differentially expressed. BgRatio: Ratio of geneset to the total number of genes in the background universe. P.adjust: P.value for enrichment of term adjusted for false discovery. geneID: List of differentially expressed genes associated with that GO term. Count: Number of differentially expressed genes associated with that GO term.

**Supplementary Table 6: Downregulated GO terms in *Nlrx1^-/-^*  vs WT in Vehicle**

As in Supplementary Table 5, but for GO terms enriched in the downregulated genes comparing *Nlrx1^-/-^* vs WT in Vehicle

**Supplementary Table 7: Upregulated GO terms in *Nlrx1^-/-^* vs WT in IFNγ+LPS**

As in Supplementary Table 5, but for GO terms enriched in the upregulated genes comparing *Nlrx1^-/-^*  vs WT in the IFNγ+LPS condition.

**Supplementary Table 8: Downregulated GO terms in *Nlrx1^-/-^* vs WT in IFNγ+LPS**

As in Supplementary Table 5, but for GO terms enriched in the downregulated genes comparing *Nlrx1^-/-^*  vs WT in the IFNγ+LPS condition.

**Supplementary Table 9. Reagents used in cell culture experiments.**

| **Reagents** | **Type** | **Company** | **Catalog number** | **Dose** |
| --- | --- | --- | --- | --- |
| IL-6 | Cytokine | PeproTech | 216-16 | 30ng/ml |
| TGFβ | Cytokine | Invitrogen | PHG9214 | 3ng/ml |
| IL-23 | Cytokine | R&D Systems | 1887-ML-010 | 10ng/ml |
| IFNγ | Cytokine | PeproTech | 315-05 | 10ng/ml |
| Anti-IFNγ | Antibody | BioLegend | 505847 | 20µg/ml |
| Anti-IL-4 | Antibody | BioLegend | 504135 | 20µg/ml |
| Anti-CD3 | Antibody | BioLegend | 100359 | 2.5µg/ml |
| Anti-CD28 | Antibody | BioLegend | 102121 | 20g/ml |
| LPS | TLR4 agonist | Millipore Sigma | L6529 | 250-500ng/ml |
| HB-EGF | Growth factor | PeproTech | 100-47 | 5 ng/mL |

**Supplementary Table 10. Primary antibodies used for immunofluorescent staining and Western blotting**

| **Antibody** | **Source** | **Company** | **Catalog number** | **Dilution** |
| --- | --- | --- | --- | --- |
| β-Actin | Mouse | Cell Signaling Technology | 3700S | 1:5000 |
| Brn3a | Rabbit | Synaptic Systems | 11003 | 1:1000 |
| Brn3a | Guinea pig | Synaptic Systems | 411 004 | 1:1000 |
| CD3 | Rabbit | Agilent Technologies | A045201-2 | 1:200 |
| GFAP | Chicken | Invitrogen | PA1-10004 | 1:2000 |
| Iba1 | Rabbit | Wako Chemicals | 019-19741 | 1:500 |
| Iba1 | Mouse | Millipore Sigma | MABN92 | 1:500 |
| Myelin basic protein (SMI99) | Mouse | Biolegend | 808401 | 1:2000 |
| Neurofilament light chain | Rabbit | Encor Biotechnology | RPCA-NF-L-ct | 1:1000 |
| NLRX1 | Rabbit | ProteinTech | 17215-1-AP | 1:2000 |
| PSD95 | Rabbit | Abcam | ab182581 | 1:1000 |
| Phospho-c-Jun | Rabbit | Cell Signaling Technology | 3270S | 1:1000 |
| Sox9 | Rabbit | Millipore Sigma | AB5535 | 1:1000 |
| Synaptophysin1 | Chicken | Synaptic Systems | 101006 | 1:500 |

**Supplementary Table 11. Reagents used for flow cytometry**

| **Reagent** | **Type** | **Company** | **Catalog number** | **Dilution** |
| --- | --- | --- | --- | --- |
| Zombie NIR | Far-red viability dye | BioLegend | 423106 | 1:2000 |
| CD45 | Spark Blue 550 | BioLegend | 103166 | 1:400 |
| CD45 | BV605 | BioLegend | 103140 | 1:400 |
| CD4 | eFluor450 | eBioscience | 48-0041-82 | 1:400 |
| CD11b | BV510 | BioLegend | 101263 | 1:400 |
| IA/IE | BV785 | BioLegend | 107645 | 1:400 |
| Clec12A | APC | BioLegend | 143406 | 1:400 |
| Ly6G | BV650 | BioLegend | 127641 | 1:400 |
| Ly6G | PE | BD Bioscience | 551461 | 1:400 |
| IL-17 | FITC | eBioscience | 53-7177-81 | 1:200 |
| IFNγ | PerCp-Cy5.5 | BioLegend | 505822 | 1:200 |

**Supplementary Table 12: RT-qPCR Primer Nucleotide Sequences**

| **Gene** | **Forward Primer** | **Reverse Primer** |
| --- | --- | --- |
| *Actb* | CTGGATGGCTACGTACATGG | ACCTTCTACAATGAGCTGCG |
| *AIF-1* | GTCCTTGAAGCGAATGCTGG | CATTCTCAAGATGGCAGATC |
| *C1qb* | CCAACGCGAACGAGAACTAT | GTGGTCACCTGGAAGGTGTT |
| *C3* | ACAACCTCGAGGAATCATGC | CCTTGTCTAGCCGGACATTC |
| *Ccl2* | TTAAAAACCTGGATCGGAACCAA | GCATTAGCTTCAGATTTACGGGT |
| *Ccl20* | GCCTCTCGTACATACAGACGC | CCAGTTCTGCTTTGGATCAGC |
| *Cxcl1* | GCTTGAAGGTGTTGCCCTCAG | AGAAGCCAGCGTTCACCAGAC |
| *GAPDH* | ACTCCACTCACGGCAAATTC | TCTCCATGGTGGTGAAGACA |
| *GFAP* | TCCTGGAACAGCAAAACAAG | CAGCCTCAGGTTGGTTTCAT |
| *HPRT* | CTCATGGACTGATTATGGACAGGAC | GCAGGTCAGCAAAGAACTTATAGCC |
| *Ifna* | ATTGGCTAGGCTCTGTGCTTT | AGGGCTCTCCAGACTTCTGC |
| *Ifnb* | TTGCCATCCAAGAGATGCTC | TCAGAAACACTGTCTGCTGG |
| *Il1a* | CGCTTGAGTCGGCAAAGAAAT | CTTCCCGTTGCTTGACGTTG |
| *Il1b* | CCTTCCAGGATGAGGACATGA | TGAGTCACAGAGGATGGGCTC |
| *Il6* | TGATGGATGCTACCAAACTGG | TTCATGTACTCCAGGTAGCTATGG |
| *Lcn-2* | CCAGTTCGCCATGGTATTTT | CACACTCACCACCCATTCAG |
| *Ligp1* | GGGGCAATAGCTCATTGGTA | ACCTCGAAGACATCCCCTTT |
| *Nos2* | GCAAACATCACATTCAGATCCC | TCAGCCTCATGGTAAACACG |
| *PSMB8* | CAGTCCTGAAGAGGCCTACG | CACTTTCACCCAACCGTCTT |
| *Tnf* | TGTGCTCAGAGCTTTCAACAA | CTTGATGGTGGTGCATGAGA |
