# Supplementary figures and images for: NLRX1 limits inflammatory neurodegeneration in the anterior visual pathway
Source: J Neuroinflammation. 2025 Jan 28;22:21. doi: 10.1186/s12974-025-03339-0 (PMC11773851; doi:10.1186/s12974-025-03339-0)

Uncorrupted plots and images

Figure 1.

NLRX1

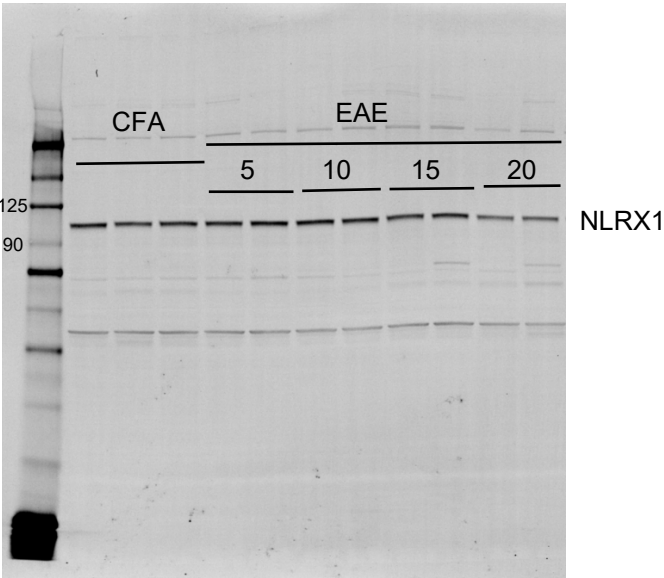

$\beta$ -Actin

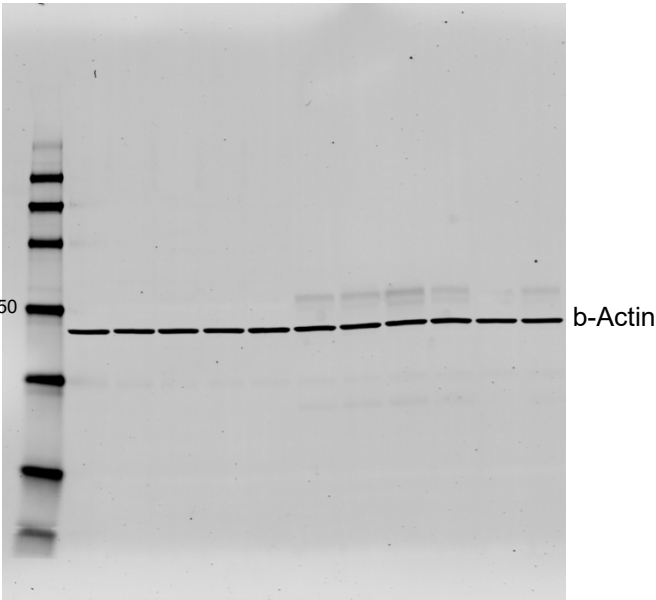

**Suppl. Fig. 1**

NLRX1

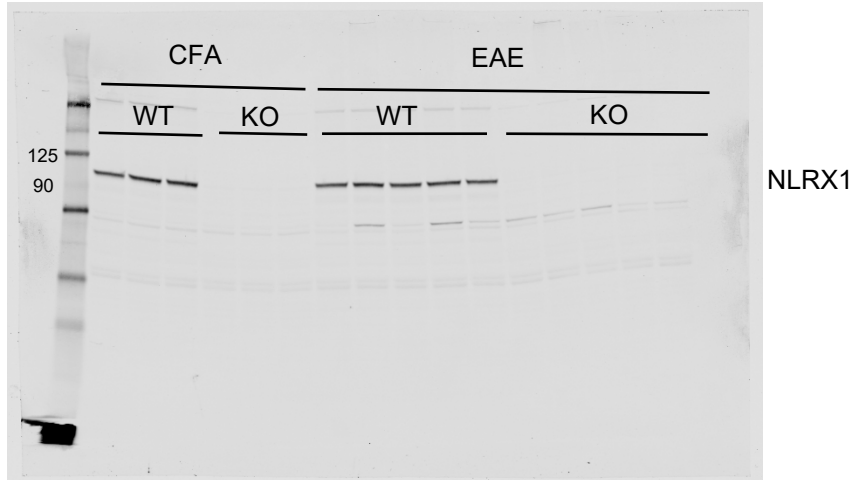

$\beta$ -Actin

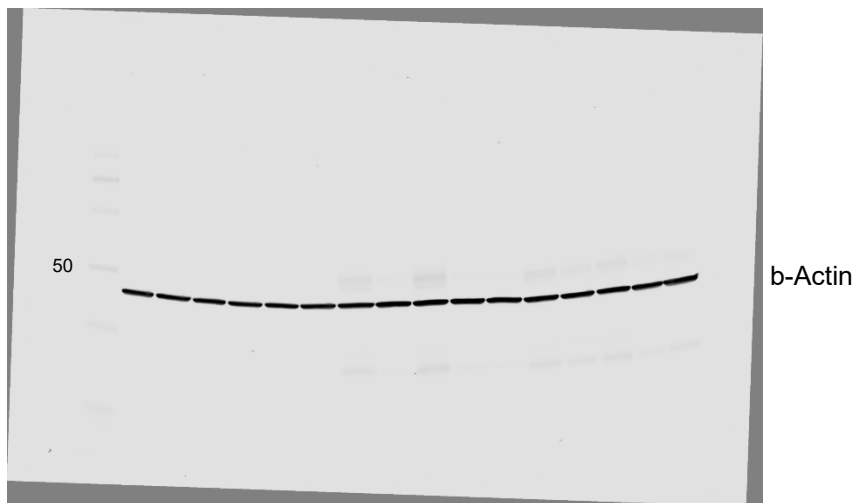

Supplement: Supplementary file 3 — Supplementary Material 3 [file 12974_2025_3339_MOESM3_ESM.pdf]
